# Comprehensive whole genome hybrid assembly unravels genes and secretome of *Albugo candida* (Indian isolate), causing white rust disease in *Brassica juncea*

**DOI:** 10.3389/ffunb.2026.1756853

**Published:** 2026-09-04

**Authors:** Samridhi Mehta, Laxmi Awasthi, Rakhi Tomar, Yashwant Kumar Yadava, Mahesh Rao, Yuvaraj Iyyappan, Bishnu Maya Bashyal, Jameel Akhtar, Ram Charan Bhattacharya, Pramod Kumar Rai, Ashish Kumar Gupta

**Affiliations:** 1 ICAR- National Institute for Plant Biotechnology, New Delhi, India; 2 ICAR-Indian Agricultural Research Institute, New Delhi, India; 3 ICAR-National Bureau of Plant Genetic Resources, New Delhi, India; 4 ICAR- National Institute of Biotic Stress Management, Raipur, Chhattisgarh, India

**Keywords:** *Albugo candida*, *Brassica juncea*, high molecular weight DNA, nanopore, secretome, whole genome assembly

## Abstract

White rust disease elicited by the biotrophic pathogen *Albugo candida* is a leading cause of yield losses in oilseed *Brassica* crops, especially in the widely cultivated *Brassica juncea*. In the present investigation, the high-quality draft genome of a virulent Ac2V race of *A. candida* infecting *B. juncea* was sequenced using Illumina and Nanopore technologies. The raw data were assembled into a genome of 36.88 Mb with 415 scaffolds and N50 = 301.91 kb. The variant analysis showed 124,974 single nucleotide polymorphisms (SNPs) with an average density of 3.3 SNP per kb genome against the Ac2VPB reference assembly. Approximately 24.29% of the genome was constituted of repetitive elements, including 1,039 SSRs. A total number of 13,715 coding genes were revealed in the genome with an average distribution of 359.03 genes per Mb. Out of these predicted genes, 11,556 were annotated based on sequence homology and 355 were predicted as effectors with no transmembrane domain and N-terminal signal peptide. The annotation of 355 effectors revealed that 141 of them had homologs, while the remaining 214 were novel. Depending on the conserved motifs, these effectors were characterized, and their role in pathogenesis was established through qPCR in *B. juncea*. Additionally, phylogenetic analysis through average nucleotide identity revealed a similarity of 99.6% between the Canadian and Indian Ac2V isolate. Furthermore, 62 new contigs were identified in the Indian isolate that showed no sequence similarity to the Canadian isolate, suggesting variation within the race. Altogether, the present work provides genomic resources and framework for the dissection of this pathogen, which will refine our understanding of the *Albugo*–*Brassica* interaction.

## 1 Introduction

*Albugo candida* (Pers.) O. Kuntze belonging to the class Oomycetes is a highly destructive biotrophic pathogen causing white rust disease and a significant yield-limiting factor among widely cultivated vegetable and oilseed brassicas (Verma et al., 2015). The disease is named after the development of characteristic creamish white pustules filled with zoosporangia on the abaxial surface of leaves in susceptible hosts, eventually coalescing into larger pustules as the disease progresses (Saharan et al., 2014). Moreover, the infection can potentially disseminate systemically to the inflorescence resulting in its abnormal development, called the “stagheads”.

In general, obligate biotrophy results in basic compatibility with a specific host due to a coevolution and codependent process (Cooper et al., 2008; Thines, 2014). Consequently, this adaptive evolution ensures efficient exploitation of the host species, while leading to an incompatible reaction in the non-host plants (Raman et al., 2012). However, *A. candida* comprises a diverse array of physiological races that can infect multiple hosts within the Brassicaceae family (Pound and Williams, 1963; Kole et al., 2002; Panjabi-Massand et al., 2010). Among the races reported to date, race 2 (Ac2V) results in a significant yield loss of 20%–60% in the economically important oilseed mustard, *Brassica juncea* worldwide (Gupta et al., 2018). In addition to race 2, race Ac7 predominantly confined to *B. rapa* has also been known to infect some genotypes of *B. juncea* (Petrie, 1988). Apart from these races, additional 22 physiological races have been documented, affecting over 200 species spanning 63 genera (Jouet et al., 2019).

The inter-relation between genetic diversity and pathogenicity differences has also been observed among the geographically distinct *A. candida* isolates. Jouet et al. (2019) did a comprehensive phylogenetic analysis of *Albugo* spp. collected from different hosts and geographical regions and observed differences in the clustering of the Ac2V isolates from India and Canada. Moreover, RAPD and diversity analyses based on ITS and COX sequences have also shown great variation in the geographically differentiated isolates of race 2 (Yadav et al., 2019). The differences in the virulence pattern of *A. candida* isolates collected from the three different agroclimatic regions of India, namely, Hisar, Pantnagar and Samastipur, have also been reported based on the biochemical variation caused by the infection in *B. juncea* (Chatterjee et al., 2022). These varying levels of aggressiveness among the isolates for most of the pathogens are probably due to the differences in their genetics and molecular characteristics, resulting in distinct secretome repertoires among these isolates (Gupta et al., 2022).

Typically, biotrophs of the fungal class Oomycetes are highly adaptive with specialized and extensive secretomes. These secreted proteins are responsible for the suppression of host defense, thus ultimately facilitating the disease progression by exploiting the host physiology (Mapuranga et al., 2022). Nevertheless, the Ac2V secretome is documented to be two-thirds the size of the secretome of another oomycete pathogen, *Phytophthora infestans* (Raffaele et al., 2010). In contrast to the extensive secretome expansions observed in *Phytophthora* and *Hyaloperonospora* genomes, the *A. candida* secretome displays a comparatively limited number of gene families. Furthermore, many predicted genes in the *A. candida* genome show little sequence similarity to those of other oomycetes. This notable sequence divergence suggests specific adaptations in *A. Candida*, which could also describe its generalist nature. Therefore, many pathological and molecular studies are underway to comprehend the pathogenic variability in *A. candida*. As the pathogen is evolving rapidly, there is a need for advanced genomic studies to understand the genetic variability. To date, whole genome sequencing has been reported for race 4, isolate AcNc2 infecting *A. thaliana* from Norwich (UK); AcEm2, which was collected from the wild brassica, *Capsella bursa-pastoris* from Kent (UK) (Borhan et al., 2008); race 9, isolate AcBoT and AcBoL from *B. oleracea* in Lincolnshire (UK) (Kemen et al., 2011); and race 2, isolate Ac2VRR (Links et al., 2011) and Ac2VPB (Furzer et al., 2022), which infects *B. juncea*, collected from Canada.

Recent developments in sequencing techniques and advanced bioinformatics tools have facilitated rapid and comprehensive analysis of genomic data, revealing insights into virulence-associated genes, secretome, regulatory and signaling cascades, epigenetic factors that impact host–pathogen interactions, and genetic diversity. Profiling of SSRs among the isolates also aids in understanding the evolutionary relationships across species. In addition, exploring single nucleotide polymorphisms (SNPs) and functional variants altering phenotypes provides opportunities to investigate pathogens’ association with virulence. Therefore, to support ongoing efforts toward the *Brassica* improvement program with respect to white rust disease management, the present study reports a high-quality draft genome of the *A. candida* race 2 Indian isolate, which is highly pathogenic to *B. juncea* cultivated under Indian agroclimatic conditions. Additionally, we predicted the genome-wide distribution of SNPs, transposable elements (TEs), and SSRs to gain insights into genetic diversity and genome organization. Furthermore, prediction of the effector repertoire of *A. candida* through secretome analysis and annotation can provide insights into the pathogenesis of this fungal pathogen. The genomic assets generated through this study will help in formulating effective control strategies for combating this destructive pathogen in Indian mustard (*B. juncea*).

## 2 Materials and methods

### 2.1 Purification and maintenance of *A. candida* inoculum

Seeds of the susceptible *B. juncea* cv. Pusa Jaikisan acquired from ICAR-National Institute for Plant Biotechnology (NIPB), India were sown in sterile soil and maintained at 15 h of light with the daytime temperature of 16 °C and 14 °C at night in growth chambers. One-month-old plants were subsequently inoculated with *A. candida* zoosporangia to establish a fresh infection and maintain the pathogen. For inoculum preparation, the zoosporangial powder was scraped using a gelatin capsule from the pustules on the infected leaves of *B. juncea* collected from the fields of ICAR-Indian Agricultural Research Institute (IARI), Delhi, India. The sporangia were then homogenized in sterile double-distilled water (sporangia count ~ 2×10^4^) and incubated at 8 °C for 1.5 h and kept for another 10 min at room temperature to facilitate the release of zoospores (Mehta et al., 2023). The plants were drop inoculated (10 μL) using a surface sterilized pipette and kept in the dark at 14 °C for 24 h. Subsequently, the inoculated plants were transferred to the growth chamber for disease development. After 12–14 days post inoculation (dpi), zoosporangial powder was collected from the rust pustules on heavily infected leaves in a sterile gelatine capsule for DNA extraction.

### 2.2 Morphological and microscopic analysis of *A. candida*

The infected leaves were collected at 12 dpi in Carnoy’s solution for destaining up to 24 h, followed by rehydration with distilled water, for microscopic analysis. The rehydrated leaves were stained using aniline blue and observed with a fluorescence microscope (Axiolab4, Carl Zeiss, Germany). For other structures, a suspension of fungal inoculum from infected leaves was mounted with 60% lactic acid and observed under brightfield. Sporangial sizes (in μm) from five sporangia per pustule were recorded (total of 10 pustules, *n* = 50) at 40× magnification. The pustule size (in mm) was also recorded by measuring the length and width of 50 randomly selected pustules using a scale. The color of the pustules was visually assessed from the infected samples.

### 2.3 Genomic DNA extraction

A modified CTAB-based protocol was established for isolating high-molecular-weight DNA from the biotrophic pathogen *A. candida* yielding high-quality DNA suitable for various downstream applications. Zoosporangial powder from a single gelatin capsule was taken in a 2-mL microcentrifuge tube and was ruptured by dry beating for 30 s using glass beads (1–2 mm) under cryogenic conditions, followed by DNA extraction through the CTAB method. Ruptured spores were homogenized in CTAB buffer and the vials were incubated at 65 °C for 30–40 min. These tubes were then centrifuged for 5 min at 10,000 rpm, to separate supernatant from the debris. Tubes were mixed with 20 µL of RNase (20 mg/mL; G biosciences) and kept in a water bath for 10 min at 65°C. Subsequently, a mix of phenol:chloroform:isoamyl-alcohol (Merck) was added in equal volumes and invert mixed. These tubes were then centrifuged at 13,000 rpm for 10 min to separate the sample mix into three layers. The top aqueous layer was carefully pipetted into a fresh vial, to which double the volume of chilled 100% isoamyl-alcohol was added and mixed gently. The vials were kept at −20 °C for 30 min to precipitate the DNA and then centrifuged for 20 min at 13,000 rpm under cool temperature (4 °C). The DNA pellet was allowed to air dry under laminar flow to remove any residual ethanol and subsequently suspended in 1× TE buffer. DNA concentrations and purity were measured with a Qubit 4 fluorometer (Thermo Fisher Scientific, USA), and the integrity was checked on 0.8% agarose gel (**Supplementary Figure 1**). Furthermore, to check for any other contamination in the DNA sample, ITS amplification was performed using the ITS1 (forward) and ITS4 (reverse) primers (White et al., 1990). The amplicon was separated on 2% gel and eluted using the PureLink gel extraction kit (Invitrogen, USA) for Sanger sequencing.

### 2.4 Library preparation and sequencing

The genome was sequenced on two platforms, NovaSeq 6000 (Illumina, California, USA) for short reads and MinION MklB (Oxford Nanopore Technologies, UK) for long reads. Library preparation for Nanopore was done using the Ligation Sequencing kit (SQK-LSK109) as per the manufacturer’s protocol, which included DNA repair and end prep for adapter attachment followed by purification with AMPure XP beads. For Illumina sequencing, randomly fragmented DNA (200–400 bp) were processed for adapter ligation, 3′ adenylation, end repair, and PCR amplification. Following magnetic bead purification, the Qubit 4 fluorometer was used to qualify library (Thermo Fisher Scientific, USA), and insert length was checked on agarose gel (2%). Libraries that qualified were sequenced on both the platforms (Centyl Biotech, India). Furthermore, base calling of the Nanopore Signal Dataset was performed using the guppy (5.0.1) high-accuracy base calling model in order to derive the Fastq sequences.

### 2.5 Bioinformatics and statistical analysis

#### 2.5.1 Data processing

The Illumina reads were trimmed for bases of low quality, adapters, and poly X tails using Fastp v0.23.2 (Chen et al., 2018). Reads were retained after trimming if the average base quality was greater than Q30. For the Nanopore data, sequences were filtered with a length threshold of 1,000 bp and passed through PoreChop v0.2.4 (Wick et al., 2017) to remove adapters and to split chimeric reads if any. Furthermore, the quality of the raw sequences from nanopore was checked through LongQC (Fukasawa et al., 2020). The Nanopore and Illumina data were further subjected to contamination screening, since it is possible that many micro-eukaryotes can harbor host genetic content or form relationships with other species or kingdoms of life. *Albugo* genomes from NCBI were downloaded and used for mapping with minimap2 2.24-r1122 for Nanopore reads (Li, 2018) and BWA-MEM for Illumina reads (Li and Durbin, 2009). For the Illumina dataset, reads that mapped pairwise were extracted, while, for Nanopore, all the mapped reads were considered. The processed and contamination free nanopore reads were then corrected with NECAT (Chen et al., 2021).

#### 2.5.2 Genome assembly

The processed reads were directly assembled into contigs with Canu v2.2 (Koren et al., 2017). The assembly was further pruned to remove duplicate contigs and to resolve them into alternative haplotypes using purge dups v1.2.6 (Guan et al., 2020). For scaffolding, ntLink-Longstitch v1.3.4 was used (Coombe et al., 2023). Later, Samba from MaSuRCA v4.0.9 (Zimin and Salzberg, 2022) was employed, keeping a matching length of minimum 2,000 bp and a maximum overhang of 500 bp, to fill the gaps. Finally, the scaffolds were polished iteratively with five rounds of Racon v1.4.3 (Vaser et al., 2017) using Nanopore mapped reads, and subsequently with 4 rounds of POLCA from MaSuRCA using Illumina reads to improve the base accuracy of the genomic scaffolds.

#### 2.5.3 Gene prediction and annotation

Prior to gene prediction, the genome was repeat-masked using Red v2.0 (Repeat Detector) (Girgis, 2015) to mask the false gene models arising due to TEs, low complexity regions, and other interspersed repeats. Four publicly available RNA-seq datasets (ERR4395359, ERR4395360, ERR4395361, and ERR4395362) were aligned to the repeat-masked genome using HISAT2 v2.2.1 to identify exon–intron boundaries and provide transcript evidence for gene prediction. In addition, protein homology evidence was obtained from 31 reference Oomycota proteomes downloaded from UniProt. Gene prediction was performed using BRAKER2 (Bruna et al., 2021), integrating both RNA-seq alignments and protein homology evidence. BRAKER2 was executed with the fungus training option using GeneMark-ES (Ter-Hovhannisyan et al., 2008), where ProtHint (Bruna et al., 2020) first generated putative gene models from the reference proteomes, followed by GeneMark-ES predictions that were subsequently used to train AUGUSTUS (Stanke et al., 2006) for final gene model prediction.

The predicted gene models were annotated functionally by eggNOG emapper v2.1.8 (Cantalapiedra et al., 2021) and InterProScan v5.55-88.0 (Jones et al., 2014) to predict protein domains through Pfam; putative pathways and metabolic reactions were annotated through Kyoto Encyclopedia of Genes and Genomes (KEGG) (Kanehisa and Goto, 2000), GO (Ashburner et al., 2000), and COG (Tatusov et al., 2000) databases; while carbohydrate-active enzymes (CAZymes) were annotated through the CAZyDB (carbohydrate-active enzymes database) (Drula et al., 2022). For the prediction of transfer RNAs (tRNAs), tRNAscan-SE with eukaryotic parameters was used (Chan et al., 2021). The rRNAs were predicted using RNAmmer (Lagesen et al., 2007), and for annotation of small nuclear RNAs, homology search against the Rfam database was done (Kalvari et al., 2021).

#### 2.5.4 Identification of transposable elements, repeated sequences, and variant analysis

For the *de novo* estimate of repeated sequence in scaffolds, RepeatModeler2 (Flynn et al., 2020) was used. Subsequently, RepeatMasker v4.1.5 (Smit et al., 2023) was used to identify the type of repeat sequence along with their location and frequency within a genomic segment, utilizing the database generated from RepeatModeler2 as the reference library. SSRs were predicted through the MicroSAtellite Identification (MISA) tool (Thiel et al., 2003) to find the type of motif families and their frequency in the genome. The SNPs and indels were predicted by read alignment of the present assembly with the available genome sequences of *A. candida* Ac2VRR and Ac2VPB using the BWA-MEM aligner. The output file resulting from the alignment was then converted to a BAM file using samtools v0.1.19 (Danecek et al., 2021). For variant calling, the mpileup program from bcftools (Danecek et al., 2021) was used at a read depth of 10×.

#### 2.5.5 Secretome and effector identification

**Figure 1:**
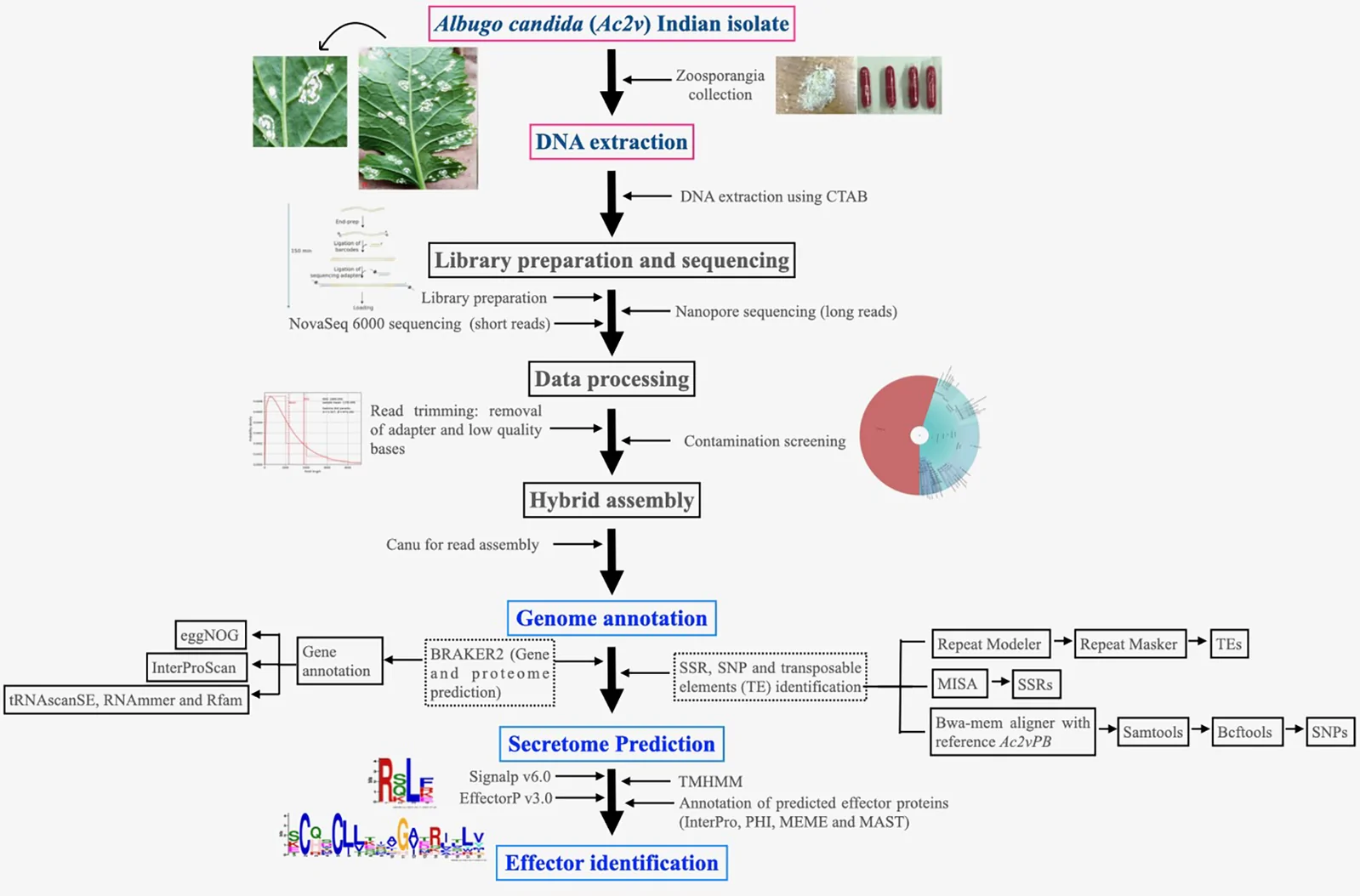
Schematic overview of the whole genome sequencing and downstream bioinformatic analysis performed for the *A. candida* (Ac2V) Indian isolate.

Transeq (EMBOSS) (Madeira et al., 2024) was utilized to translate the predicted gene sequences into protein sequences, which were then used as query for the command line-based SignalP v6.0 program (Teufel et al., 2022) to discover the putative secretory proteins on the basis of N-terminal signal peptides. Subsequently, TMHMM v2.0 (Krogh et al., 2001) was used to further shortlist the secretory proteins with no transmembrane domains. The candidate secretory proteins (CSPs) were then filtered through EffectorP-3.0 (Sperschneider and Dodds, 2022) to identify and characterize them into cytoplasmic and apoplastic effectors. The candidate effectors were annotated with Pfam (Mistry et al., 2020), CAZy, and PHI-base (Urban et al., 2022) through BLAST v2.16.0-based homology search (*E*-value ≤ 1e−05) (Zhang et al., 2000). Furthermore, effectors without any significant hits were classified based on the motifs within the first 30–100 amino acids using the command line MEME suite (*E*-value < 10) (Bailey et al., 2015) (**Figure 1**).

#### 2.5.6 Quantitative expression of the predicted effectors

The mature leaves of 60-day-old *B. juncea* (cv. Pusa Jaikisan) plants grown in three independent pots with four plants each were inoculated with *A. candida* Delhi isolate under controlled conditions following the standardized inoculation technique (Mehta et al., 2023). The RNA was isolated from the leaf samples collected after 1, 7, and 12 days post-infection. The cDNA was prepared using the verso cDNA synthesis kit as per the manufacturer’s protocol (Thermo Fisher Scientific). Real-time qRT-PCR was conducted using Eva Green SYBR mix (G Biosciences), with the CFX Opus 96 Real-Time detection system (Bio-Rad, USA) using gene-specific (**Supplementary Table 7**). The relative gene expression was calculated using 2^−ΔΔCt^, where ubiquitin was taken as an internal transcript control (Chen et al., 2014; Attard et al., 2014). Each gene expression level was quantified using three biological and three technical replicates each. Two-step qRT-PCR reaction conditions were as follows: 95 °C for 10 min, followed by 40 cycles of 95 °C for 15 s, and 60 °C for 1 min. This was followed by a Melt curve step consisting of 95 °C for 15 s, followed by 60 °C for 1 min, and 95 °C for 15 s.

#### 2.5.7 Similarity-based clustering and phylogenomic analysis

The nine representative genomes of *Albugo* species, along with other oomycete pathogens as listed in **Table 1**, were downloaded from the NCBI (data accessed on 2 January 2024). Average nucleotide identity (ANI) of the draft genome was calculated using FastANI v1.34 (Jain et al., 2018) with alignment fraction considered to account for genome coverage during comparisons. Furthermore, phylogenomic relationships among selected oomycete genomes were inferred using a phylogenomic approach based on conserved single-copy orthologs identified using the BUSCO v5.6.1 (Manni et al., 2021) “stramenopiles_odb10” dataset. The single-copy BUSCO genes present in all 21 genomes were extracted, and their protein sequences were aligned individually using MAFFT v7.525 (Katoh and Standley, 2013) and concatenated into a super matrix comprising 87 orthologs. Maximum likelihood phylogenetic analysis was performed using IQ-TREE v2.4.0 (Minh et al., 2020) with automatic model selection and 1,000 bootstrap replicates. Visualization was carried out using an online server-based tool iTOL v7.5.1 (Letunic and Bork, 2024).

**Table 1:** List of the representative genomes of the fungal pathogens of class oomycetes.

Pathogen species	Assembly accession
*Albugo candida* (Ac2VPB)	CAJNAR010000001.1
*A. candida* (Ac2VRR)	JAHTBM010000001.1
*A. candida* (Ac7v)	LGTM01000001.1
*A. candida* (AcEm2)	JZXB01000001.1
*A. candida* (AcEx)	LGTN01000001.1
*A. candida* (AcNc2)	CAIX01000001.1
*A. laibachii* (AlNc14)	CACSLJ010000001.1
*A. candida* (AcBot)	CAIV01000001.1
*A. candida* (AcBol)	CAJG01000001.1
*Peronospora belbahrii*	CAKKTJ010000001.1
*Hyaloperonospora parasitica*	JAPPWB010000001.1
*Peronosclerospora sorghi*	CM047580.1
*Plasmopara halstedii*	NW_020186923.1
*Phytophthora parasitica*	KI669561.1
*Phytophthora infestans*	DS029320.1
*Phytophthora sojae*	NW_009258115.1
*Pythium aphanidermatum*	KE463865.1
*Pythium oligandrum*	JAHRIK010000001.1
*Pythium vanterpoolii*	JAADVD010000001.1
*Bremia lactucae*	CM033803.1

## 3 Results

### 3.1 Morphological and microscopic analysis of *A. candida*

**Figure 2:**
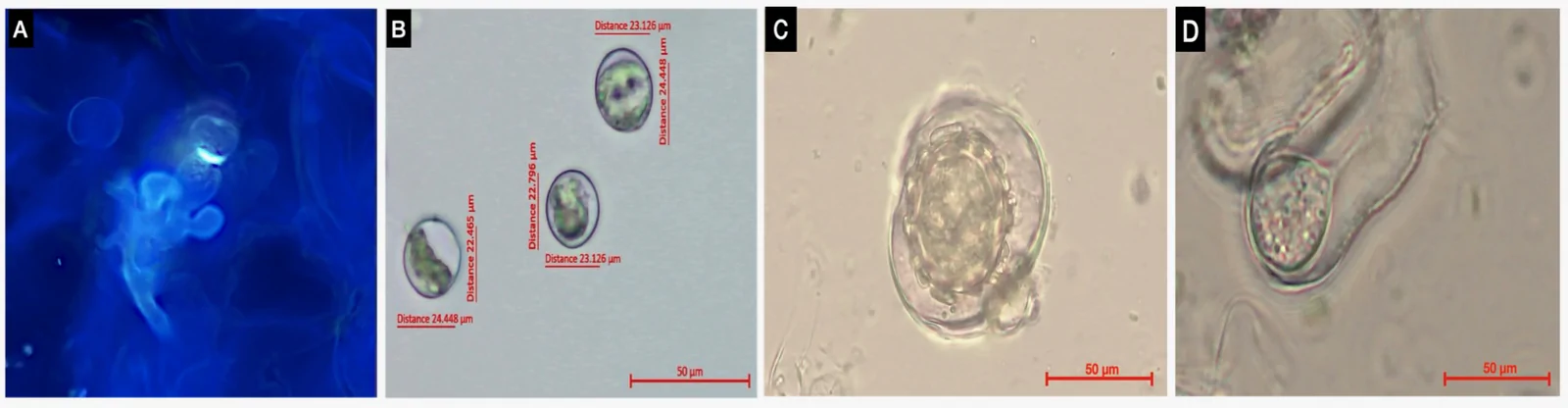
Figures showing microscopic images of *A. Candida*. **(A)** Club-shaped conidiophore. **(B)** Sporangia. **(C)** Oospore with verrucose indentations. **(D)** Globose haustoria with a narrow stalk.

Leaves collected from white rust-infected *B. juncea* plants were observed for disease development. The pustules appeared on the abaxial surface, irregularly distributed, whitish to creamish in color, with a length ranging from 2.5 to 4.5 mm (average, 3.6 mm; *n* = 50) and a diameter of 1–4 mm (average, 2.6 mm; *n* = 50). Light microscopy of the infected leaves revealed branched hyphae that were regularly or irregularly swollen. Some intercellular hyphae developed into unbranched, thick walled, cylindrical or clavate sporogenous hyphae that formed a long chain of sorus filled with numerous sporangia arranged in basipetal succession (**Figure 2A**). Sporangia appeared hyaline and globose with diameter ranging from 22.5 to 27.5 μm (average, 22.7; *n* = 50) (**Figure 2B**). Furthermore, the sexual oospores or oogonia were thick walled, pale yellow in color, globose, and tuberculate with blunt ridges on the wall surface that are often confluent or branched (**Figure 2C**). The haustoria, which function as exchange sites for nutrient uptake and transfer of fungal virulence factors such as effectors, were mostly globose in shape and surrounded by a thick sheath with a narrow stalk (**Figure 2D**).

### 3.2 Genome sequencing, assembly, and BUSCO analysis

The purity of the high-molecular-weight DNA isolated from the zoosporangial mass of the *A. candida* (Ac2V) pathogen”Delhi” isolate was confirmed by sequence homology of the ITS region via PCR amplification followed by Sanger sequencing (NCBI accession: PX472090). The contaminant-free DNA was then subjected to complete sequencing on the Oxford Nanopore (ONT) platform for long reads and on the Illumina HiSeq platform for short reads. The ONT and paired-end Illumina libraries generated a total of 6.3 and 4.5 GB, respectively.

**Figure 3:**
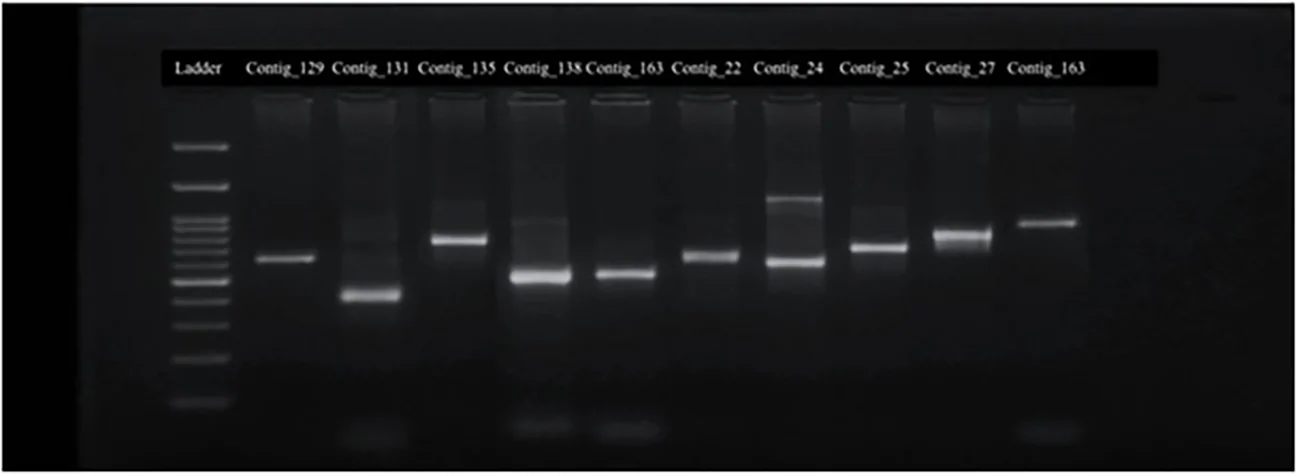
Gel image showing the amplification of newly identified contigs in the *A. candida* Ac2V race isolate”Delhi”.

The raw data were then filtered based on the quality score and trimmed, and adapter sequences were removed. Error correction was performed to obtain reads representing at least 40× genomic coverage. The corrected reads were then assembled into contigs using Canu v2.2. The resulting contigs were scaffolded using ntLink implemented in Longstitch. Gap filling was subsequently carried out using the Samba module from MaSuRCA v3.4.2. Finally, the Illumina reads were used for polishing with POLCA to improve the base accuracy of the genomic scaffolds and gap filling. The aforementioned assembler statistics resulted in 415 scaffolds with an N50 of 301.91 kb, an L50 of 39, and a hybrid assembly of 36.88 Mb of *A. candida* (**Table 2**). The assembly is longer than SOLID-based Ac2VRR (34.56 Mb) and the Illumina-based AcNc2 assemblies; however, it is slightly smaller than the PacBio-based Ac2VPB assembly (38.96 Mb) (Links et al., 2011; Furzer et al., 2022). The contigs were also evaluated for homology search using NCBI BLASTn v2.16.0 (*E*-value threshold: 1e-10). Out of 12,961,225 bp aligned, 1.28497e+07 were found to match the Ac2VPB assembly with an overall identity of 99.1395. While most of the contigs matched with the Ac2vPB assembly, 62 contigs were identified with no significant hits (**Supplementary Table 6**). To validate the new findings, some contigs (contig_129, contig_131, contig_135, contig_138, contig_163, contig_22, contig_24, contig_25, and contig_27) were taken at random for experimental validation. Contig-specific primers were designed for PCR, and amplification of the expected product confirmed the presence of these sequences in the genome (**Figure 3**). Conversely, reciprocal BLAST with Ac2VPB assembly as query was also performed with the present contigs. All the scaffolds present in the Ac2VPB assembly showed sequence similarity to one or more contigs from the Delhi isolate assembly with a total of 5,165,957 aligned bp, of which 5,115,252 were matching with a 99.0185 identity.

**Table 2:** Statistics of the draft genome assembly of the *A. candida* Delhi isolate.

Statistics	*A. candida* assembled genome
Quantitative analysis of genome assembly	
Size of assembled genome	36.88 Mb
Total number of scaffolds in assembly	415
N50 scaffold length	301.91 kb
Longest scaffold length	1.03 Mb
L50	39
GC (%)	43.44%
No. of genes predicted	13,715
Qualitative analysis of assembly	
BUSCOs complete (percentage)/fragmented (percentage)/missing (percentage) (Metaeuk with Stramenopiles_odb)	100%/0%/0%
BUSCOs complete number (percentage)/fragmented number (percentage)/missing number (percentage) (Augustus with Stramenopiles_odb)	97%/1%/2%
BUSCO for proteins	
BUSCOs complete number (percentage)/fragmented number (percentage)/missing number (percentage) (Stramenopiles_odb)	99 (99%)/0 (0%)/1 (1%)
BUSCOs complete number (percentage)/fragmented number (percentage)/missing number (percentage) (fungi_odb)	433 (57.1%)/50 (6.6%)/275 (36.3%)

These 62 contigs were further searched for homology against other *Albugo* genomes, which showed 25 contigs with significant hits. Out of these, 14 contigs aligned with AcNc2 assembly (2.79%–72.20% coverage with 75.64–100 identity) and 2 with Ac7v (6.62%–28.72% coverage and 80.906–87.33 identity), while 4 contigs aligned with *A. laibachii* (7.92%–67.29% coverage with 79.431–81.91 identity). These three assemblies came from *Albugo* pathogen isolated from *A. thaliana.* Additionally, four contigs showed hit with *A. candida* assembly Bol (8.47%–17.42% coverage with 83.43–95.98 identity) and one with Bot (20.06% coverage with 97.6 identity) both isolated from infected *B. oleracea* (**Supplementary Table 8**). Furthermore, the 62 contigs were checked for the presence of any predicted proteins. A total 22 proteins were found on 11 contigs, all of which were LTR retrotransposons and mobile genetic elements with reverse transcriptase domains, integrase core domain, retrotransposon structural proteins, retroviral protease, chromodomain-associated retroelements, nucleic acid binding retroelement motif, retrotransposon replication machinery, and DNA transposon-associated proteins.

**Figure 4:**
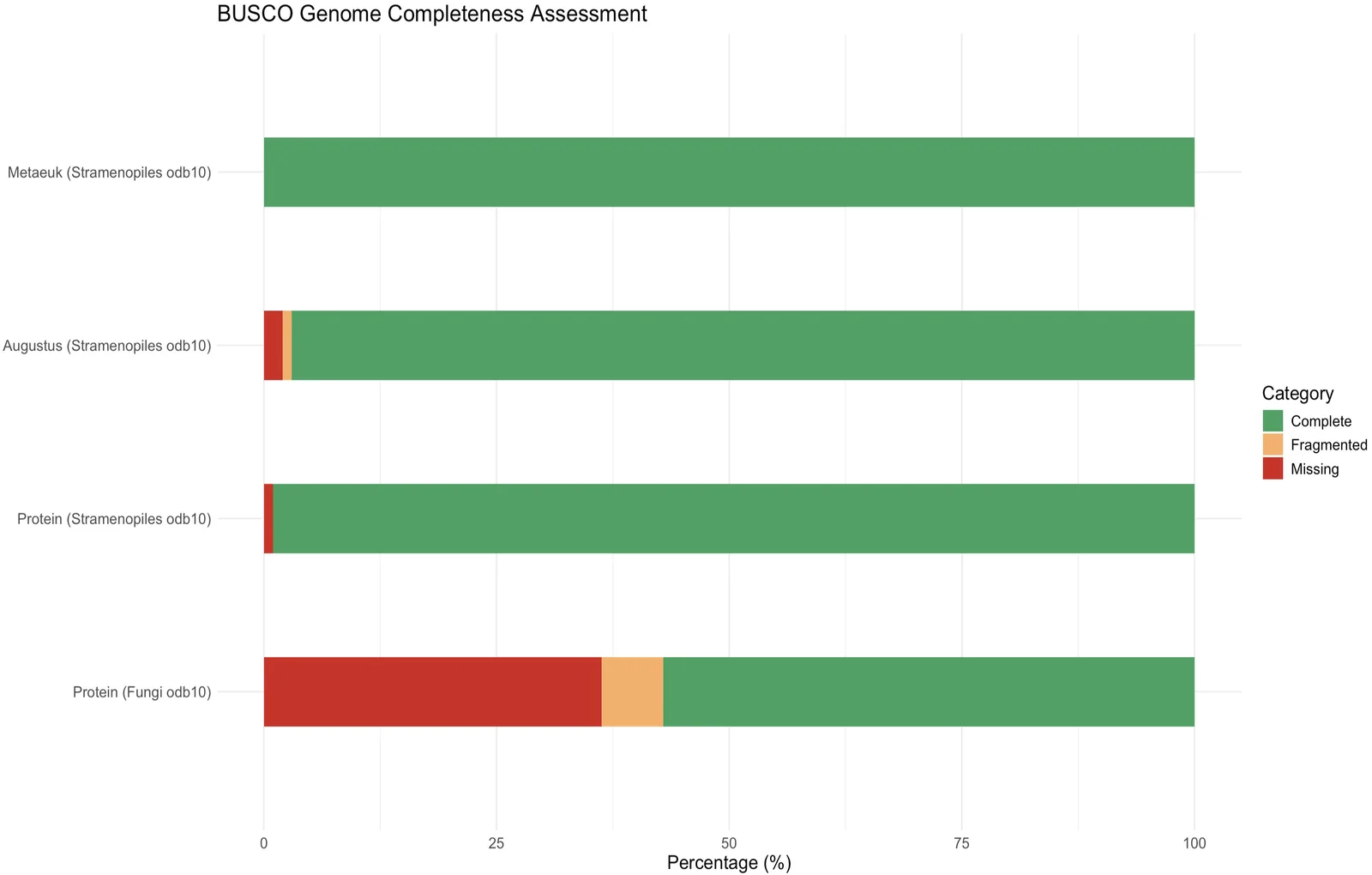
BUSCO analysis to evaluate the completeness of the Ac2V_Delhi genome assembly using MetaEuk and Augustus gene prediction approaches with the Stramenopiles_odb10 dataset, as well as protein-based searches against both Stramenopiles_odb10 and Fungi_odb10 lineage datasets.

The quantitative assessment of *A. candida* hybrid genome assembly and annotation completeness was carried out using the BUSCO Stramenopiles_odb10 dataset. The draft assembly showed high completeness, with 97% complete, 1% fragmented, and 2% missing BUSCOs using Augustus-predicted genes, while analysis using the MetaEuk pipeline recovered 100% complete BUSCOs, indicating a near-complete representation of conserved orthologs in the assembly. Furthermore, BUSCO analysis of the predicted proteins from the assembly showed 99% of complete BUSCOs for the Stramenopiles_odb10 database and 57.1% for the fungi_odb10 gene lineage database. This reduction is expected due to the evolutionary divergence between fungi and oomycetes, highlighting the importance of lineage-specific datasets for accurate genome completeness estimation (**Figure 4**, **Table 2**).

### 3.3 Bioinformatics analysis

#### 3.3.1 Transposable elements, repetitive sequences, and variant analysis

TEs are the major mechanistic drivers of genome evolution; therefore, whole-genome data were analyzed for a better understanding of the transpositional landscape of the *A. candida* pathogen (**Table 3**). The *A. candida* genome harbors both Class I and Class II TE families, with repeat elements occupying only 24.29% of the genome assembly. Long terminal repeat (LTR) retroelements were the most abundant TEs, representing 7.87% of the assembly, followed by Long interspersed nuclear elements (LINEs), which constitute 7.3% of the genome. Among the LTR elements, Copia/TY1 (5.6%) was more prevalent than Gypsy/DIRS1 (2.01) and retroviral elements (0.27). Additionally, the repeat sequences comprised DNA transposons (3.19%), low-complexity repeats (0.02%), small RNAs (0.39%), simple repeats (0.23%), and unknown sequences (5.65%).

**Table 3:** Types of transposable elements identified in the *A. candida* genome.

Types of transposable elements	Number of elements	Length occupied (bp)	Percentage of sequence (%)
Retroelements	5,219	5,697,587	15.45
SINEs	274	101,513	0.28
Penelope	0	0	0
LINEs	1,732	2,691,482	7.3
CRE/SLACS	0	0	0
L2/CR1/Rex	0	0	0
R1/LOA/Jockey	0	0	0
R2/R4/NeSL	0	0	0
RTE/Bov-B	134	90,188	0.24
L1/CIN4	673	1,083,305	2.94
LTR elements	3,213	2,904,592	7.87
BEL/Pao	0	0	0
Ty1/Copia	2,460	2,066,099	5.6
Gypsy/DIRS1	696	740,442	2.01
Retroviral	57	98,051	0.27
DNA transposons	1,535	1,175,672	3.19
hobo-Activator	0	0	0
Tc1-IS630-Pogo	468	505,134	1.37
En-Spm	0	0	0
MULE-MuDR	1,052	667,095	1.81
PiggyBac	0	0	0
Tourist/Harbinger	0	0	0
Other (Mirage, P-element, Transib)	0	0	0
Rolling circles	0	0	0
Unclassified	5,954	2,084,549	5.65
Total interspersed repeats		8,957,808	24.29
Small RNA	357	143,706	0.39
Simple repeats	1,295	85,889	0.23
Low complexity	164	7,310	0.02

**Figure 5:**
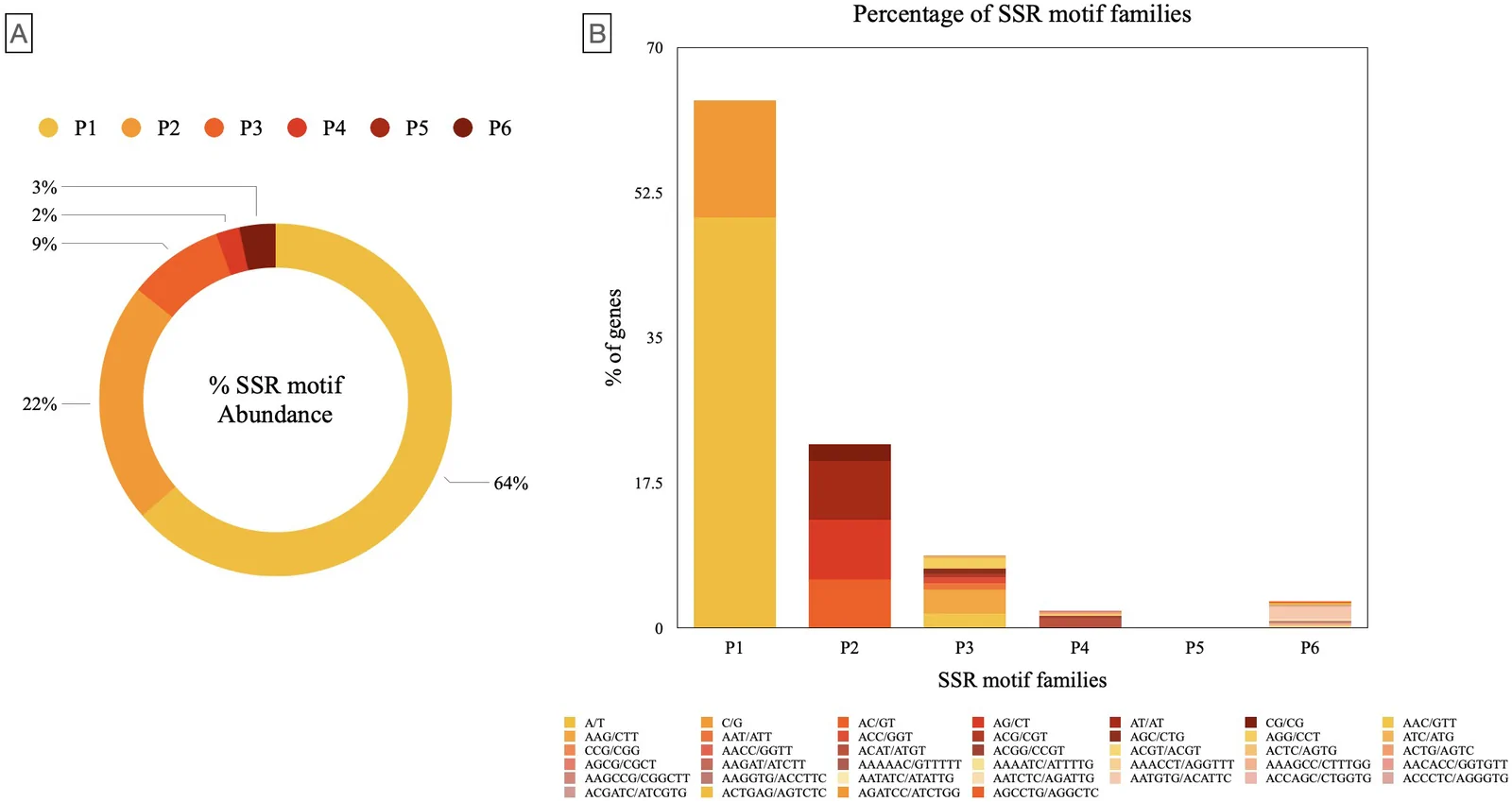
Classification and distribution of SSRs identified in the *A. candida* genome. **(A)** Proportions of SSRs with different motif types. P1: mononucleotide repeats; P2: dinucleotide repeats; P3: trinucleotide repeats, P4: tetranucleotide repeats; P5: pentanucleotide repeats; P6: hexanucleotide repeats. **(B)** Frequency of distribution of different SSR motif families.

A total of 1,039 SSRs were identified with a relative abundance of 28.16 SSRs/Mb of the Delhi isolate genome. The most frequent motifs were mononucleotides (63.6%) followed by dinucleotides (22.1%), trinucleotides (8.7%), tetranucleotides (2.1%), pentanucleotides (0.1%), and hexanucleotides (3.3%). The genome sequence also yielded 108 compound SSRs. The percentage frequency of SSRs based on the nucleotide size revealed that SSRs composed mostly of smaller repeats, while SSRs with large repeats represented a smaller percentage. The distribution of SSR types is heterogeneous, particularly for mono- and dinucleotides. In mononucleotides (P1), A/T repeats (77.7%) were the most frequent motif in the entire genome, followed by G/C (22.2%). Among dinucleotides (P2), the highly distributed motifs were AG/CT (32.6%), AT/AT (31.7%), and AC/GT (26.5%), while in trinucleotides (P3), motifs AAG/CTT (32.9%) and AAC/GTT (19.7%) were the most abundant. Among tetranucleotides (P4), ACAT/ATGT (54.5%) was copious, and in pentanucleotides (P5), the only representative sequence was AAGAT/ATCTT, and in hexanucleotides (P6), motif AATGTG/ACATTC (41.1%) was mostly prevalent (**Figure 5**). The present assembly was also compared with the Ac2VPB and Ac2VRR assemblies for variant analysis. A total of 124,974 variants were identified in the genome against Ac2VPB, out of which 124,923 were SNPs and 51 were InDels, while 92,444 SNPs and 52 InDels were found against the Ac2VRR assembly, highlighting the variations among the isolates of *A. candida*.

#### 3.3.2 Gene prediction and functional annotation

**Figure 6:**
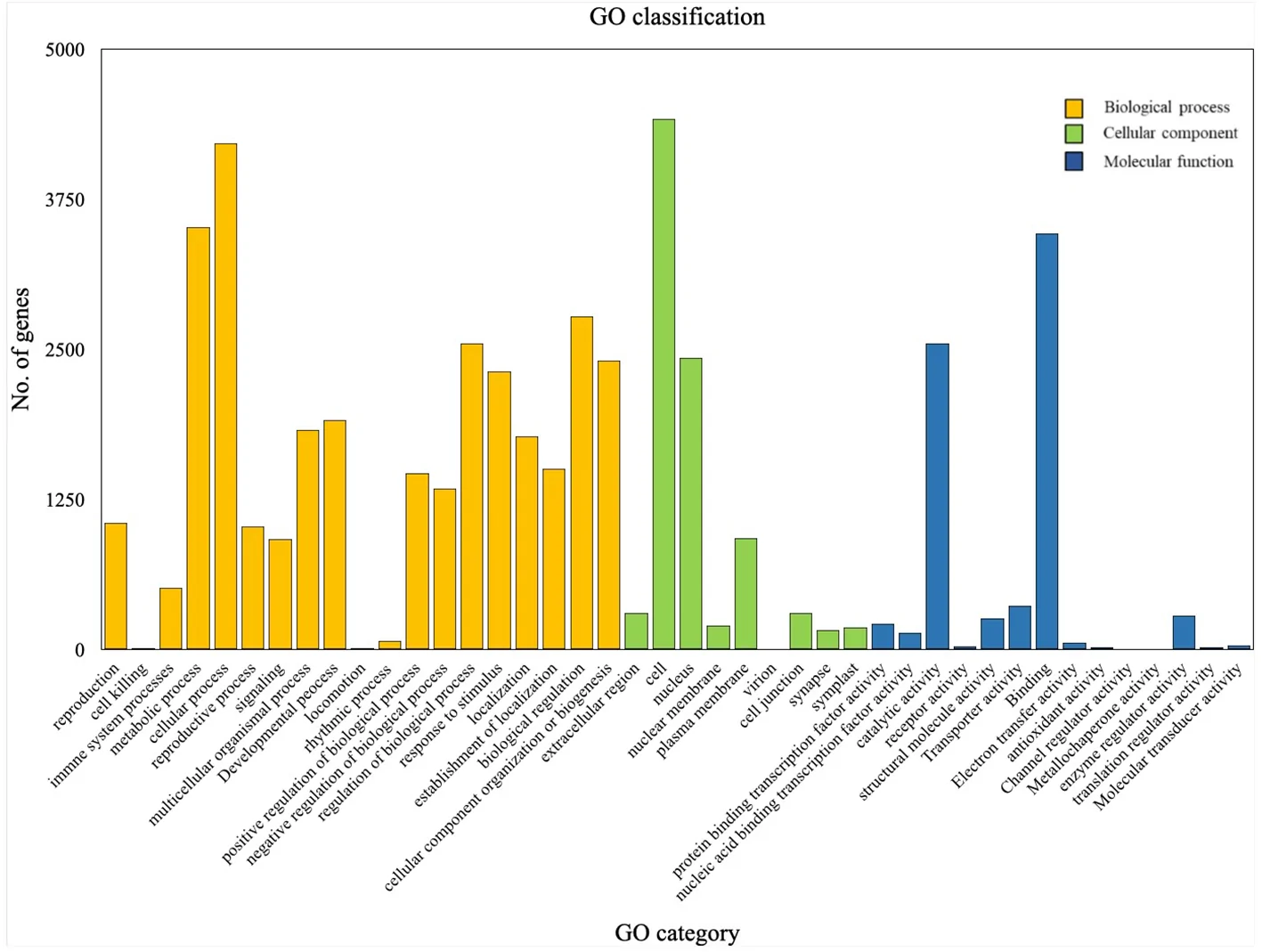
Gene Ontology (GO) annotation of the *A. candida* Delhi isolate genome showing the functional classification of predicted genes into the three major GO categories: Biological Process, Molecular Function, and Cellular Component.

Scaffolds arising from the primary assembly were repeat-masked, and comprehensive gene predictions were carried out using the BRAKER2 program, followed by functional annotation using eggNOG-EMAP v2.1.8 and InterProScan v5.55-88.0 to predict protein domains, GO terms, and putative pathways. A total of 13,715 genes were predicted in the Indian isolate of *A. candida*, which is comparable to the predicted genes in the Ac2VPB assembly (13,073), but surprisingly fewer than Ac2VRR (15,826). The present annotation, while containing fewer genes, is more complete and less fragmented, as evaluated by BUSCO analysis. Out of the predicted 13,715 genes, 11,556 genes were annotated with GO, COG, KEGG, CAZy, and Pfam databases, accounting for 84.2% of the total predicted genes (**Table 4**). tRNAscan-SE, Rfam, and Rnammer were used to predict tRNA, snRNA, and rRNA, respectively. Results showed that the genome contained 314 tRNAs, 22 snRNAs, and 66 ribosomal RNAs represented by 48 copies of the 8S subunit, while only 6 copies of the 28S and 18S subunit each. GO analysis for the genome revealed that the top five enriched GO terms were cell, cellular process, metabolic process, and binding, with 4,419 genes, 4,214 genes, 3,517 genes, and 3,468 genes, respectively (**Figure 6**).

**Table 4:** Functional annotation summary of the *A. candida* genome from different databases.

Database	Number of genes	Percentage
GO	4,698	34.2%
KEGG	6,357	46.3%
COG	7,358	53.6%
Pfams	9,602	70.0%
CAZy	231	1.7%
PHI	5,922	43.2%
BiGG	128	0.9%
Reactome pathway	8,783	64.0%
MetaCyc pathway	7,288	53.1%
TMHMM	2,209	16.1%

**Figure 7:**
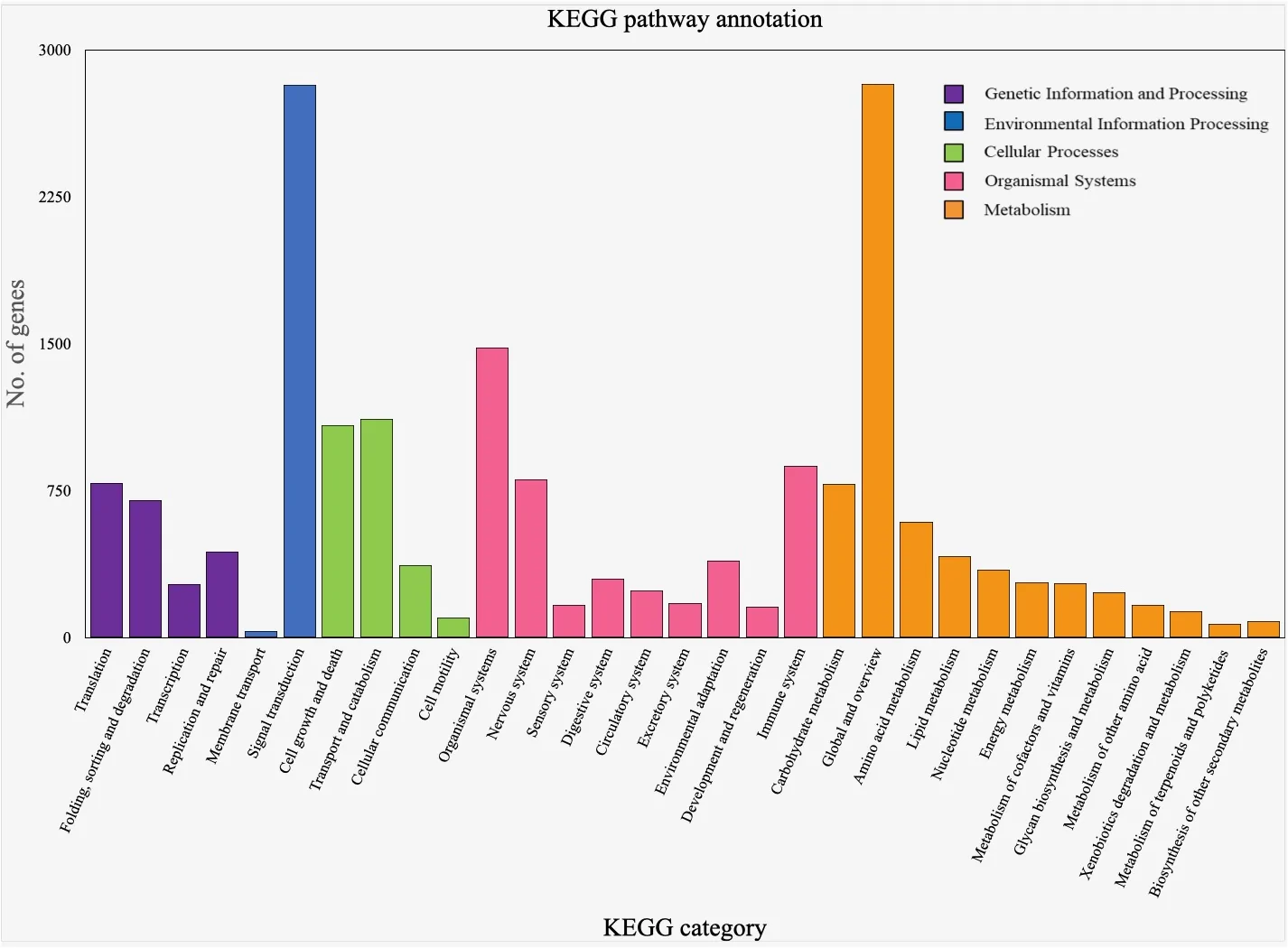
KEGG pathway annotation of the *A. candida* Delhi isolate genome illustrating the assignment of predicted genes to major metabolic and cellular pathways.

Among 31 KEGG pathways, the top five pathways were “Global and Overview”, “Signal transduction”, “Organismal system”, “Transport and catabolism”, and “Cell growth and death”, including 2,826 genes, 2,821 genes, 1,480 genes, 1,117 genes, and 1,082 genes, respectively (**Figure 7**). According to the results of gene set enrichment analysis for top KEGG pathways, a significant proportion of genes in *A. candida* Indian isolate are likely involved in metabolism, environmental information processing, and organismal systems.

**Figure 8:**
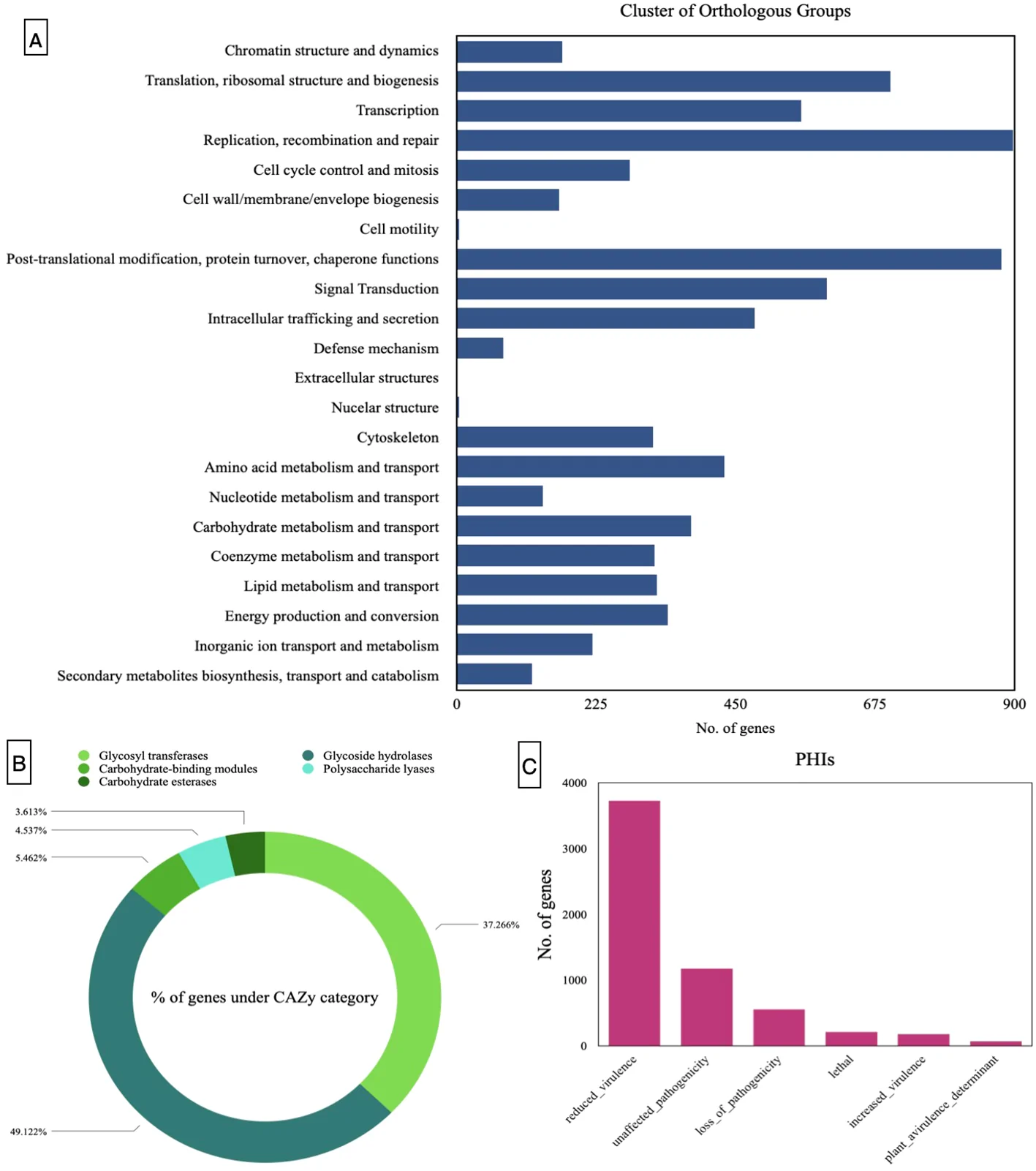
Annotation of the predicted genes from the *A. candida* “Delhi” isolate genome based on the **(A)** Clusters of Orthologous Groups of proteins (COG), **(B)** CAZys function classification of proteins, and **(C)** Pathogen–Host Interaction database.

Through COG mapping, 7,358 proteins were assigned to different COG categories (**Figure 8A**). The most gene-abundant classes in the COG groups included “Replication and repair”, with the highest number of genes (897), followed by “Post-translational modification, protein turnover, chaperone functions”, “Translation, ribosomal structure and biogenesis”, “Signal Transduction”, “Intracellular trafficking and secretion”, “Amino acid metabolism and transport”, and “Carbohydrate metabolism and transport”. These findings indicate an enriched and diverse repertoire of proteins associated with metabolic and secretory functions. These proteins perform a central role in enhancing the absorption and metabolism of nutrients from substrates, thereby facilitating infection in the host plant. The CAZy database was used on the predicted fungal genes to annotate the CAZymes. A total of 231 genes were assigned to CAZymes families, out of which the highest percent was represented by glycoside hydrolases (GHs, 49.1%), followed by glycosyl transferases (GTs, 37.3%), carbohydrate-binding modules (CBMs, 5.5%), polysaccharide lyases (PLs, 4.5%), and carbohydrate esterases (CEs, 3.6%) (**Figure 8B**). GHs are usually predominant in majority of fungal pathogens, and their function to break down glycosidic bonds is an indicative of their diverse role in fungal pathogenesis like breakdown and modification of the plant cell wall components. Especially for a biotroph like *A. candida* that depends on its host for nutrition, GHs can play a major role in cell wall manipulation allowing development of haustoria. An inclusive analysis of these CAZys can provide valuable insights into host–pathogen interactions, ultimately contributing to our understanding of disease establishment. Homology search based on BLAST results for pathogenicity-related genes revealed 9,152 genes out of 13,715 that aligned, with a threshold of *E*-value < 1e−05, to the PHI database. These pathogenesis-related proteins were predominantly categorized into six groups considering their functional characteristics and distinctive traits. Most PHI genes were categorized under reduced virulence (3,732), followed by unaffected pathogenicity (1,175), loss of pathogenicity (555), lethal (210), increased virulence (179), and least under plant avirulence determinant (71). This reflects the diverse repertoire of pathogenesis-related genes in the Indian isolate (**Figure 8C**).

#### 3.3.3 Secretome and effector diversity

Of the total 13,715 protein-encoding genes, SignalP v6.0 predicted 688 secretory proteins with signal peptides (SPs) in the N-terminal region. This repertoire of secretory proteins appears to be smaller than that identified from Ac2VRR (929 secreted proteins) and Ac2VPB (1,104 secreted proteins) isolates of *A. candida* using SignalP v3.0 (Links et al., 2011; Furzer et al., 2022). The predicted secretory proteins were then subjected to TMHMM, which identified 162 secretory proteins with one or multiple transmembrane domains. The remaining 526 secretory proteins with no transmembrane domain were then classified using EffectorP, which predicted 355 effector proteins. Among these effector proteins, 304 were categorized as cytoplasmic effectors (85.6%) and 51 were categorized as apoplastic effectors (14.4%). The predicted effector proteins were annotated against Pfam, GO, KEGG, COG, PHI, and CAZy databases to evaluate their role in pathogenesis. Annotation of the 355 effector proteins showed only 125 proteins with identity and description while the remaining 230 proteins had no significant hits and were further classified based on the motifs into RxL and CCG types (**Supplementary Table 9**). Out of the 125 annotated effectors, 64 proteins aligned to PHI-base under different categories such as reduced virulence, unaffected pathogenicity, loss of pathogenicity, hypervirulence, and avirulence effectors. Among the remaining 61 proteins, 4 were classified under CAZy as GH and GTs, while 9 were identified to have plant cell wall modification activity, namely, 3 pectate lyase, 5 polygalacturonase, and 1 cellulase activity. Additionally, three cysteine-rich secretory proteins involved in pathogenesis were also identified.

**Figure 9:**
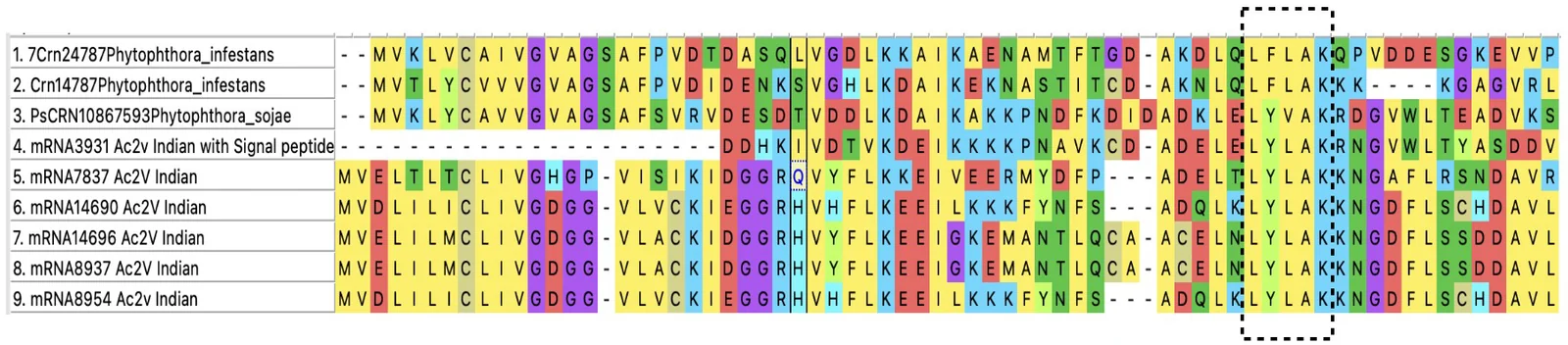
Sequence alignment to compare the CRN domain of the *A. candida* “Delhi” isolate with the *Phytophthora* spp. revealed variation in the conserved motif, where the canonical “LFLAK” domain was substituted by “LYLAK” in the *A. candida* Delhi isolate.

**Figure 10:**
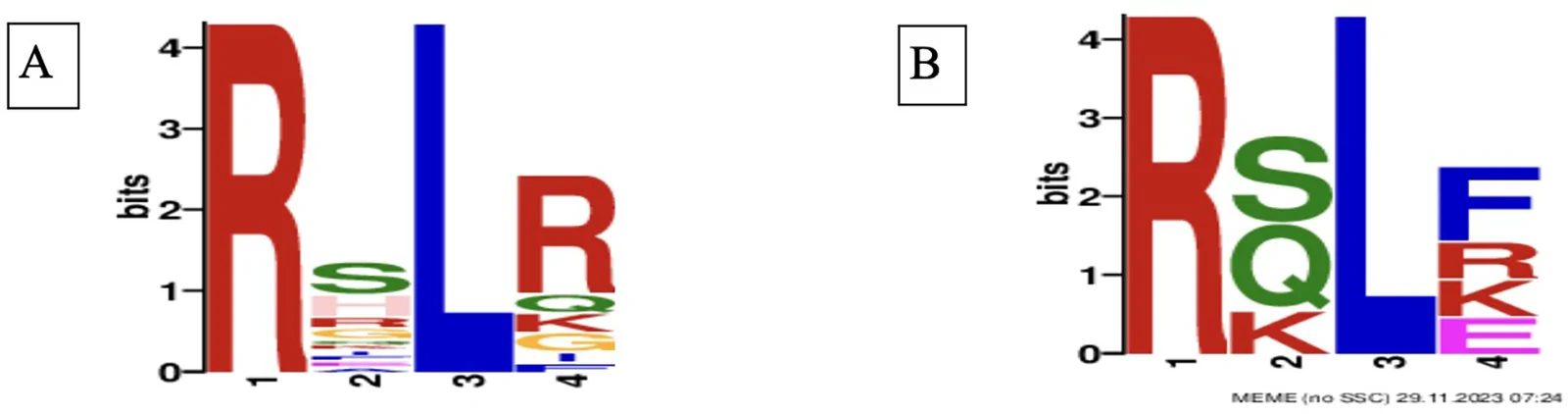
MEME output showing the comparison between the RxL motif in **(A)** Ac2VRR and **(B)** Ac2V Delhi isolate genome assemblies.

Among the crinkling and necrosis (CRN) class effectors, six proteins were identified with an amino-terminal “LYLAK” instead of “LFLAK” domain similar to the CRN effector identified in *Phytophthora sojae* (**Figure 9**) (Stam et al., 2013). Interestingly, out of the six CRN proteins, only one protein was identified as an effector, while the remaining five had no signal peptide sequence, suggesting their non-secretory nature. In addition to CRNs, six secretory proteins without transmembrane domains were annotated as elicitins, and three CBELs containing CBD and Apple domains showed 88% sequence similarity to the CBEL reported from *A. laibachii* (Kemen et al., 2011). The results also showed two effector proteins with a cellulose-binding domain following the signal peptide, but no apple domain. The search for RXLR effector candidates among the 230 unannotated effector proteins against the RXLR effector database, i.e., RdEER.hmm (Win and Kamoun, 2008) showed no hits for homology, suggesting a lack of the commonly found RxLR motif in *A. candida*. However, Links et al. (2011) reported a small group of genes that encode secreted proteins with a variant RxLR motif (Ac-RxL). Therefore, a MEME motif was built using seed sequence for “Ac-RxL” effector proteins from the Ac2VRR assembly. The motif “RSLRRSTE” was then used to search the list of putative effector proteins through MAST. In the present assembly, 13 gene models were predicted, which contained a putative sec-dependent signal peptide, lacked homology to known proteins, and carried an Ac-RxL motif R(SQK)L(FRKE) near the N-terminal (**Figure 10**).

**Figure 11:**
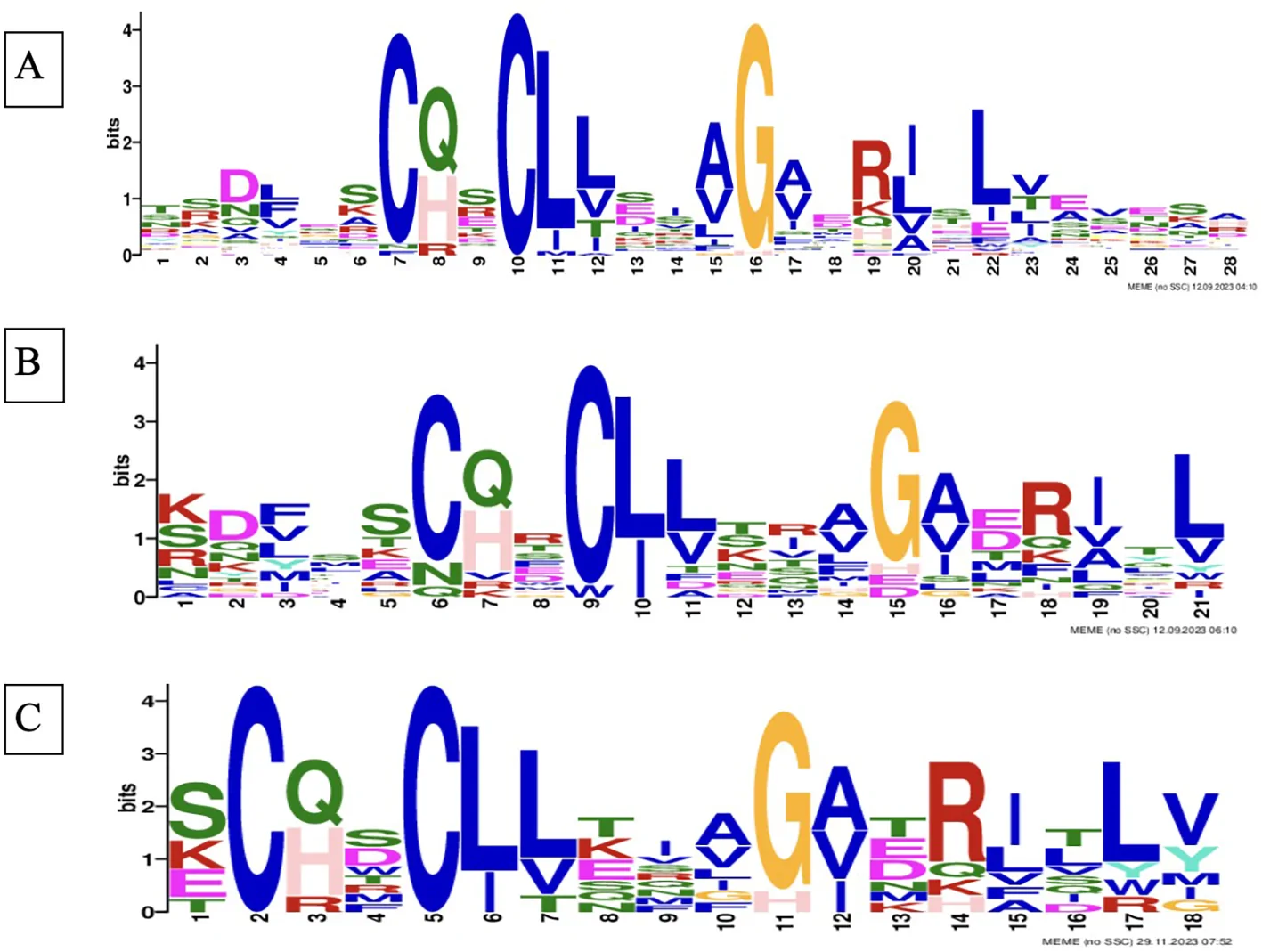
MEME output for the CCG motif showing the comparison between the **(A)** Ac2VRR, **(B)** Ac2VPB, and **(C)** Ac2V Delhi isolate genome assemblies.

CHxC effectors are another conserved effector motif commonly present in most of the oomycetes. However, Furzer et al. (2022) reported that compared with the consensus motif from the AlNc14 (*A. laibachii*) genome, the previously reported histidine residue in the CHxC motif was less conserved in Ac2VPB, but a six-amino-acid glycine after the second cysteine was highly conserved, making the consensus motif CxxCxxxxxG. They redefined the effector class as “CCGs” in *A. candida*. CCG-type effectors were also reported in the Ac2VRR assembly. Therefore, based on the sequences of previously identified CCG class effectors in the Ac2VRR and Ac2VPB genomes, a motif was generated through MEME (**Figure 11**). Using the predicted effectors as input file in MAST, a total of 15 CCG protein candidates were identified that showed no homology to other proteins, accounting for 4.2% of the total predicted effectors.

#### 3.3.4 Expression pattern of the effector candidate gene in the *A. candida* Delhi isolate

**Figure 12:**
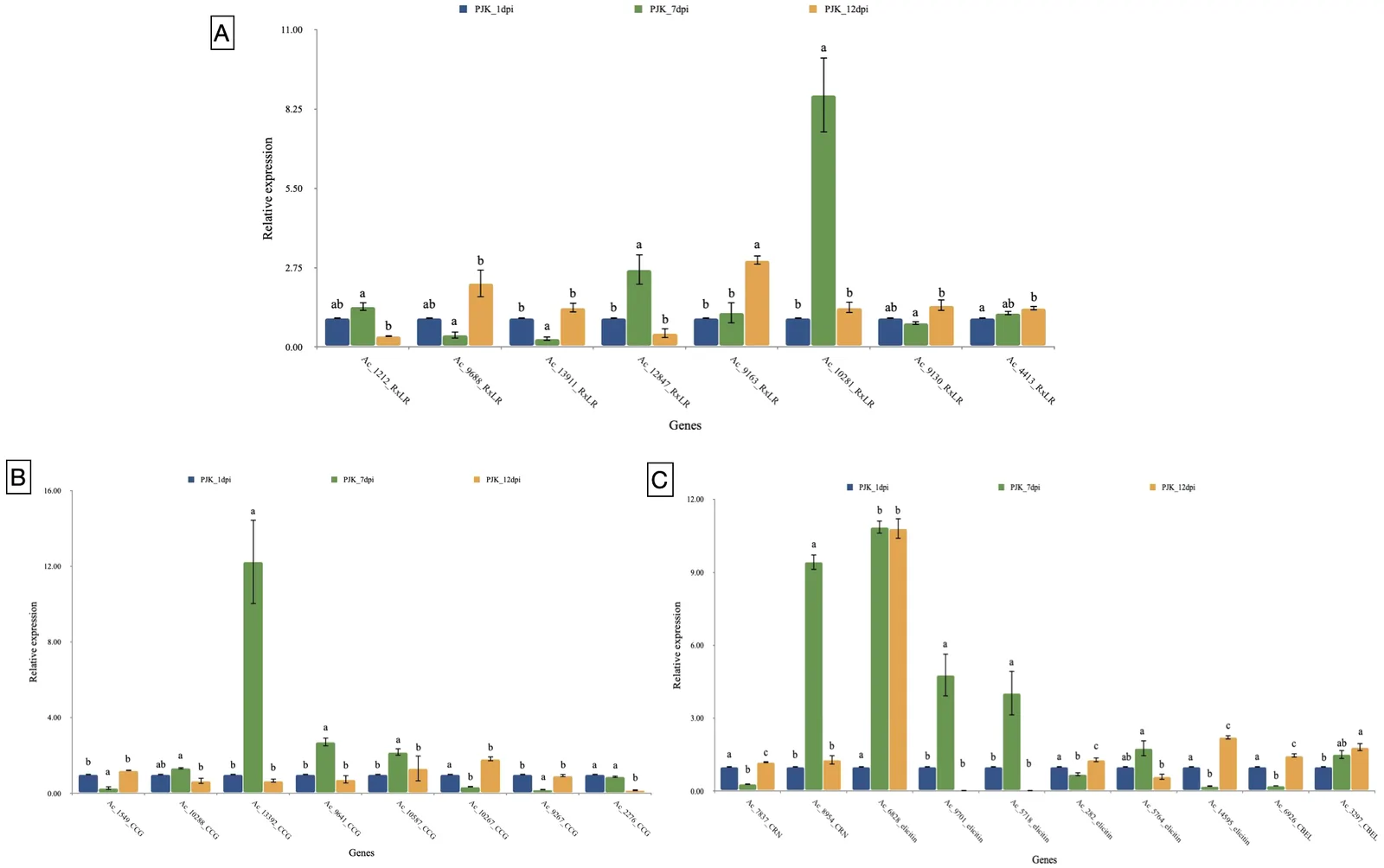
Expression analysis of the selected secretory effector genes from **(A)** CRN, elicitin, and CBEL; **(B)** CCG; and **(C)** RxL class by qRT-PCR in the *A. candida*-infected leaves of *B. juncea* at 7 and 12 days post inoculation. The expression values are the means ± SD. Statistically significant differences, indicated by distinct letters, were determined at *p* < 0.05 using Tukey’s honestly significant difference (HSD) test.

Expression analysis of predicted effector candidates was experimentally validated using the RNA extracted from the infected leaves of 60-day-old *B. juncea* cv., Pusa Jai Kisan plant at 1, 7, and 12 dpi to ascertain their expression during pathogenesis (**Supplementary Table 7**). There were three biological replicates, with each replicate corresponding to RNA extracts from pooled leaf samples of plants from each pot. Depending on the class of effectors, 43 secretory proteins were selected for the qPCR analysis, and their expression levels were quantified using the 2^−ΔΔCq^ method with ubiquitin as the reference gene. Following statistical analysis using analysis of variance (ANOVA) and Tukey’s test, 26 secretory proteins were identified as showing significant differential expression (*p*-value < 0.05). Most of them were strongly induced during late infection stages, i.e., 7 and 12 dpi, including two CRNs, six elicitins, two CBELs, eight CCG, and eight RxL. This temporal pattern suggests a strategic deployment of these effectors during the transition from colonization to sustained biotrophy. Among them, Ac_8954_CRN, Ac_6828_elicitin, Ac_9701_elicitin, Ac_5718_elicitin, Ac_13392_CCG, Ac_9163_RxL, and Ac_10281_RxL showed the highest upregulation [with mean values and SD (*n* = 3) ranging from 3.02 ± 0.16 to 12.23 ± 2.22] compared to control (i.e., 1 dpi) at different time points (**Figure 12**). This might suggest their potential role in pathogenesis and disease development.

#### 3.3.5 Genome-level distance clustering and BUSCO-based phylogenetic analysis

**Figure 13:**
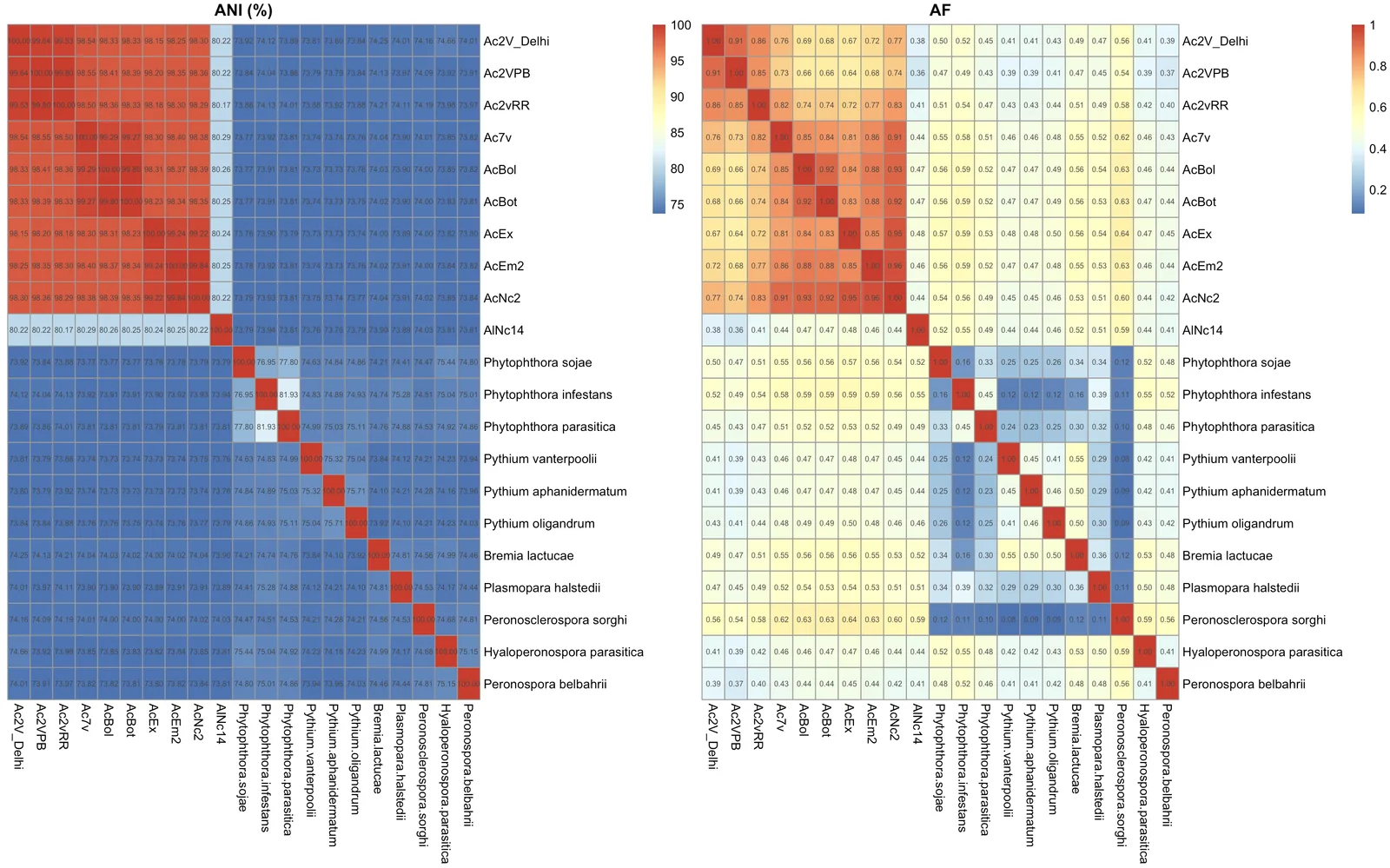
Heatmap representing average nucleotide identity (ANI) and alignment fraction (AF) between the genomes of different oomycetic plant pathogens.

Although previous studies have revealed the phylogeny and genetic diversity of *A. candida*, most of them have been based on the sequence alignment of nuclear ITS or mitochondrial COX (Choi et al., 2006; Yadav et al., 2019). In the current investigation, the genome-level similarity of the *A. candida* race Ac2V Delhi isolate phylogenetic position was elucidated using FastANI, which utilizes an alignment-free, MinHash-based algorithm. Typically, an ANI value surpassing 95% is widely accepted and is a benchmark for species classification and clustering. Conferring from the results, the ANI values within the *Albugo* genome varied from 80.17% to 99.84%. Compared to the present assembly, high ANI with assemblies Ac2VPB (99.64%, alignment coverage 0.91) and Ac2VRR (99.53%, alignment coverage 0.86) were observed, therefore clustering closely with them in the distance matrix (**Figure 13**). Among other races of the *A. candida* pathogen, this similarity ranged from 98.54% to 98.15%, with an alignment coverage of 0.76 to 0.67, while for *Albugo laibachii* race AlNc14, infecting *Arabidopsis thaliana*, the genome showed a low ANI value (80.22%, alignment coverage of 0.38), supporting its distinction at the species level. Based on sequence similarity, three distinct clusters were observed. The first cluster was composed of Ac2V Delhi, Ac2VRR, and Ac2VPB (race 2; host *B. juncea*), while the second one included Ac7v (host *A. thaliana*), AcBol, and AcBot (race 9; host *B. oleracea*), and the third cluster had AcNc2 (host *A. thaliana*), AcEx, and AcEm2 (race 4; host *C. bursa-pastoris*).

**Figure 14:**
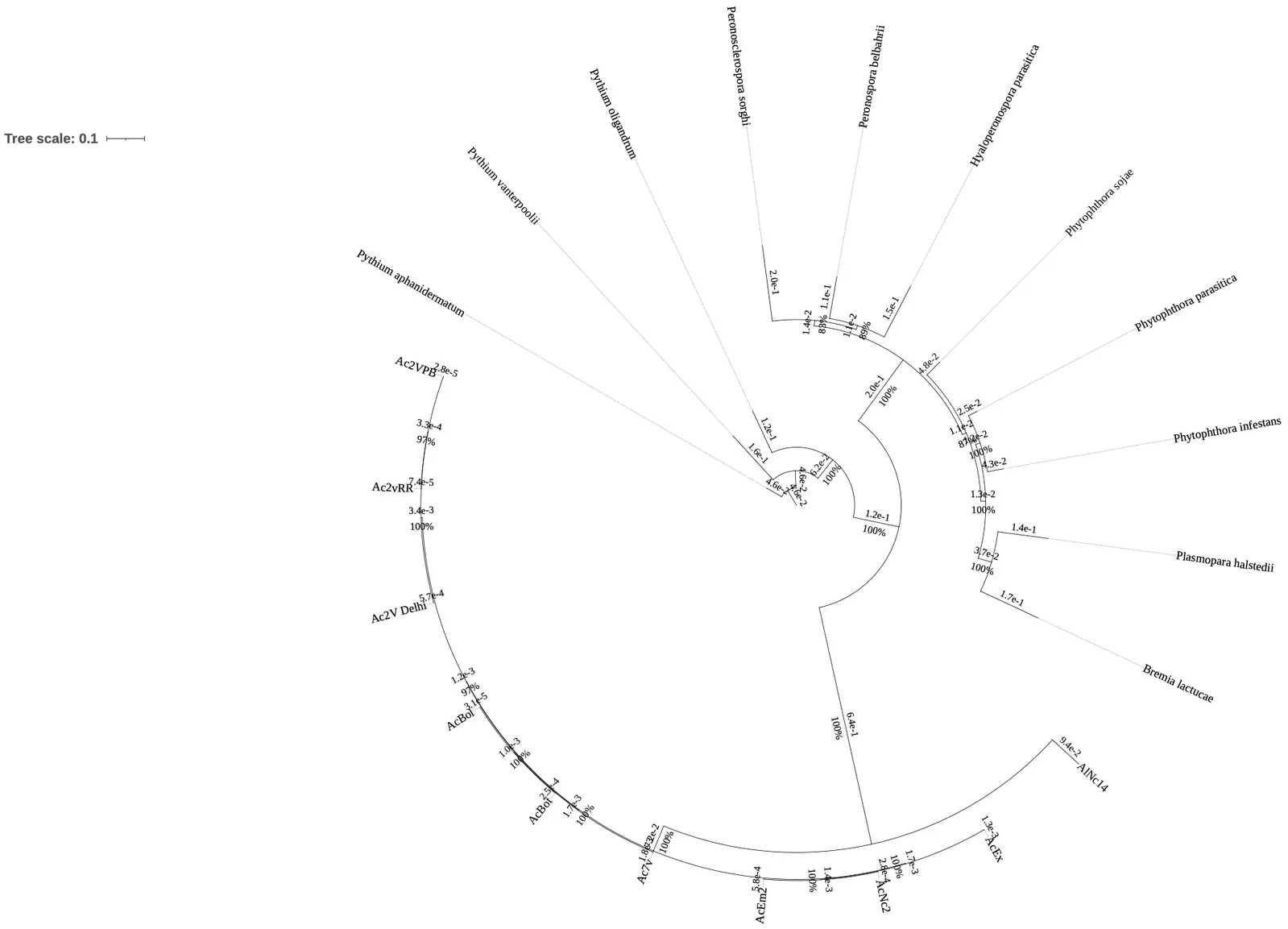
Phylogenetic tree showing Ac2v_Delhi clustering within the *Albugo* spp. clade with slight divergence indicated by branch lengths and bootstrap support values (%) shown at the nodes. The major oomycete lineages are resolved with *Pythium* species forming the outgroup.

Although ANI reflects pairwise genome similarity that provides useful insights into genomic relatedness, it does not explicitly tell phylogenetic order. Therefore, phylogenomic analysis was conducted using conserved single-copy orthologs identified with BUSCO against the stramenopiles_odb10 lineage dataset. Notably, the branching patterns based on the 87 common single-copy orthologs across the genomes suggested intra-species diversification within *A. candida*, with distinct clades representing potential lineage-specific adaptations or geographic differentiation. Branch lengths within the Ac2V_Delhi-Ac2VRR-Ac2VPB cluster were relatively shorter compared to more distantly related isolates, indicating limited evolutionary differentiation among these genomes (**Figure 14**). However, it still suggests appreciable genomic divergence among these isolates. Such variation may arise from the accumulation of mutations within conserved genes. Therefore, the clustering reflects common ancestry rather than genetic uniformity, highlighting intra-lineage diversity within *A. candida*.

## 4 Discussion

The destructive phytopathogen of *Brassica* crops, *A. candida*, causing white rust disease, is a broad host range oomycete fungus despite being a biotroph (Panjabi-Massand et al., 2010). It poses as a serious threat to the quantity and quality of oil production in widely grown Indian mustard cultivars in India and globally. Previous studies on diversity analyses have revealed substantial variation among geographically differentiated isolates of this pathogen in India (Yadav et al., 2019; Singh et al., 2021). Furthermore, the differential response of the host toward geographically distinct isolates made it necessary to dissect the various genetic elements and the genomic features of the pathogen (Chatterjee et al., 2022). Therefore, the current study provides a high-quality hybrid genome assembly of the *A. candida* Delhi isolate (Ac2V) infecting *B. juncea*, offering comprehensive insights into its genomic organization, effector diversity, and evolutionary divergence from other reported isolates. While previous assemblies, notably those from the Canadian isolates Ac2VPB and Ac2VRR (Links et al., 2011; Furzer et al., 2022), have advanced our understanding of *A. candida* biology, the present work fills an important geographic and evolutionary gap by describing an Indian variant of the same race (race 2) that has adapted to distinct agroclimatic conditions of India. The hybrid assembly using Oxford Nanopore and Illumina sequencing produced a genome of 36.88 Mb, which was 2.32 Mb larger than Ac2VRR (34.56 Mb) and 2.05 Mb smaller than Ac2VPB assembly (38.96 Mb). A total of 62 novel contigs were identified in the present study that were also validated through PCR amplification (**Figure 3**). The total length of these 62 contigs corresponded to 234,330 bp, which represents 0.63% of the total variation in the 36.88 Mb of genome. The BLAST analysis showed that out of 12,961,225 aligned bp, 12,849,700 bp matched the Ac2VPB assembly with an overall identity of 99.13%, indicating a high degree of genomic similarity between the two isolates. Furthermore, reciprocal BLAST analysis demonstrated that all Ac2VPB scaffolds showed significant matches within the Ac2V_Delhi assembly, with 5,115,252 matching bp out of 5,165,957 aligned bp at an overall identity of 99.01%. Together, these findings indicate that the Ac2V_Delhi assembly encompasses the previously reported genomic content while also containing additional putatively novel genomic regions that may represent strain-specific genetic diversity. Additionally, comparative analysis of the unmatched contigs against other available *Albugo* assemblies revealed that several of these sequences showed partial similarity with previously reported isolates infecting different hosts.

Furthermore, the functional annotation of the predicted genes on these contigs revealed that these sequences predominantly encoded LTR retrotransposons and other mobile genetic element-associated proteins. The enrichment of TE-related sequences within these unmatched contigs suggests that repetitive and mobile genomic regions may contribute substantially to structural variation in the Ac2V_Delhi isolate. Such elements are known to play a positive role in shaping genome architecture and expression of genes encoding pathogenicity factors (Moller and Stukenbrock, 2017).

Gene prediction revealed 13,715 protein-coding genes in the Indian isolate, comparable to the Ac2VPB assembly (13,073), but fewer than Ac2VRR (15,826) (Links et al., 2011; Furzer et al., 2022). However, the present annotation had 100% stramenopile BUSCOs unlike Ac2VRR with only 93%, indicating a more complete annotation despite the lower number of predicted genes.

Repeat elements are the genomic feature that enables the pathogen to rapidly adapt to the varying hosts and changing environmental conditions. In the present assembly, TEs and repeats accounted for approximately 24.29% of the genome, lower than the Ac2VPB assembly that accounted for 29% while higher than Ac2VRR with only 17% repeat content and the *A. laibachii* Nc 14 genome assembly, which had 22% of the genome’s repetitive sequence (Kemen et al., 2011). The predominance of LINEs and LTR retroelements, particularly the Copia/Ty1 family, was consistent with previous assemblies. This highlights their significant role in genome evolution, facilitating genomic rearrangements and functional diversification. Microsatellite (SSR) markers are a promising and rapid tool to decipher molecular recognition and identify the genetic differentiation among the isolates of the pathogen having differential virulence. Compared with other oomycetes, such as Phytophthora, the number of SSRs was relatively lower in the present assembly. Additionally, unlike *Phytophthora*, in which trinucleotide and hexanucleotide repeats are most prevalent, the *A. candida* Delhi isolate was enriched for mononucleotides and dinucleotides, especially A/T-rich SSR motifs (Garnica et al., 2006). Furthermore, variant analysis with the previous two *A. candida* assemblies revealed 124,974 variants against Ac2VPB, out of which 124,923 were SNPs and 51 were InDels, while against Ac2VRR, 92,444 SNPs and 52 InDels were found. This is indicative of the variation that exists between the two isolates delimited by the geographical barriers, even though they belong to the same race (race 2), therefore suggesting the highly variable nature of the *A. candida* pathogen depending on the place of origin.

Annotation results for the predicted genes showed that a higher percentage are involved in cell and cellular component development, cellular processes, and metabolic activities that serve as the building blocks for the pathogen’s rapid growth. Furthermore, the KEGG pathway analysis revealed a similar pattern of distribution, where a high number of genes were involved in signal transduction, catabolism, and transport-related pathways required for basic life functions. Interestingly, the CAZymes identified in the present study were dominated by GHs, which are pivotal in cell wall modification and penetration. Similar findings were also reported in *Phytophthora* spp., where its abundance was attributed to the complexity of carbohydrate biochemistry and the broad range of hydrolytic activities it encompasses (Ospina-Giraldo et al., 2010). The successful establishment of infection by a fungal pathogen begins with its interaction with the host, mediated through the secretion of proteinaceous effectors, hydrolytic enzymes, and various secondary metabolites. These secretory proteins play pivotal roles during the early stages of infection, promoting pathogen colonization and facilitating the host–pathogen interaction. In the present study, 526 secretory proteins were identified with a signal peptide and no transmembrane domain, which is far less than the previously reported Ac2VRR (929) and Ac2VPB (1,104) (Furzer et al., 2022). This is possibly due to difference in the pipelines and the tools used, while the previous assemblies have used SignalP v3.0, which relies on neural networks and HMMs for basic identification, and the present version SignalP v6.0 leverages on protein language models (Teufel et al., 2022).

Out of these secretory proteins, 355 were classified as effector proteins using EffectorP. While most of the effectors were involved in pathogenesis under different categories, such as reduced virulence, hypervirulence, and avirulence, effector-based on the homology with the PHI database, some of them were classified as GHs and GTs under CAZy groups. Furthermore, plant cell wall modifying activity such as pectate lyase, polygalacturonase, and cellulase was also observed among these effectors. Interestingly, cysteine-rich secretory proteins that are known to be responsible for eliciting defense response in non-host plants were also identified (Mattinen et al., 2004; Pemberton et al., 2005; Motteram et al., 2009). These can induce plant cell death, elicit strong immune responses, and are frequently associated with plant perception of PAMPs (Wang et al., 2022). Notably, among the six CRNs, only one protein was found to be a candidate effector with signal peptide, while the remaining five had no signal peptide sequence; similar results were found in *A. laibachii*, where only three members of the CRN family were identified with signal peptides while eight additional CRN-like proteins were identified where no signal peptide has been predicted (Kemen et al., 2011). This suggests the presence of both secreted and non-secreted forms of these proteins. Elicitins are another highly conserved group of secreted proteins and include members such as INF1, a 10-kDa protein from *P. infestans*. The presence of six cysteine residues is a common feature of elicitins and has been used to mine databases revealing 156 elicitins (ELI) or elicitin-like (ELL) genes from seven *Phytophthora* species (Jiang et al., 2006). In the present secretome, only six elicitins were annotated. Cellulose Binding Elicitor Lectin (CBEL) is a cell wall glycoprotein first reported from *P. parasitica* var. *nicotianae* that is responsible for eliciting a defense response in the host *Nicotiana benthamiana* (Mateos et al., 1997). CBELs from *Phytophthora* species generally have a secretion signal followed by interleaved Cellulose Binding (CBD) and Apple domains, which mediate protein–protein or protein–carbohydrate interactions. Links et al. (2011) reported two CBELs in their Ac2VRR genome. Ac2VRR-CBEL1 had a signal peptide while Ac2VRR-CBEL2 did not contain a signal peptide. However, the present study has identified three CBELs containing a signal peptide and no transmembrane domain. Overall, the present analysis on the *A. candida* secretome for pathogenicity-related proteins and potential elicitins reveals a significant reduction as compared to other oomycetes. Kemen et al. (2011) explained this reduction by suggesting that biotrophic pathogens minimize the activation of host defense responses by limiting their repertoire of secreted proteins, especially those involved in cell wall degradation. Additionally, the expression pattern of these effectors during pathogenesis was studied through qPCR. A total of 26 effectors were found to be significantly upregulated in the late infection stages (7 and 12 dpi) of disease development, which might indicate their involvement in prolonged pathogenesis, establishment of infection, and manipulation of host defense responses. Furzer et al. (2021) also observed a spectrum of expression patterns, including effectors expressing late in the infection for CCG effectors in *A. candida*. However, further functional validation through experimental studies is required to confirm their roles in immune suppression and their contribution to virulence.

Genome-level phylogenetic reconstruction based on conserved single orthologs from BUSCO and ANI confirmed that the present assembly of the *A. candida* Delhi isolate infecting *B. juncea* is a variant of race Ac2v, rather than race Ac7, which also infects the same host plant. Although the difference in ANI values among the *A. candida* isolates might not seem much, as low as 0.36% between Ac2VPB Canadian and Ac2V Delhi variant could be significant. However, one cannot rely only on ANI for resolving evolutionary order as it is appropriate for assessing overall genomic similarity and for supporting species-level relatedness. Therefore, single-copy ortholog from BUSCO was fetched for phylogenomic analysis, which also revealed subtle but evident evolutionary divergence among these two isolates. In addition, the identification of unique contigs suggests that the genome has accumulated variations, which might be the result of geographical distinction. Furthermore, the persistence of high similarity with the Canadian isolate despite spatial separation implies recent divergence and ongoing gene flow among race Ac2v populations.

The extensive data and information gathered from the current assembly of *A. candida* will also contribute to a better understanding of the racial variability within the pathogen. Deciphering the genome of Indian virulent race Ac2V of *A. candida* will also pave the way for future research in various aspects of the pathogen, including its evolutionary response to environmental variations, the biosynthesis of secretory proteins associated with its pathogenesis, and the dynamics of host–pathogen interactions. The availability of this high-quality genome assembly also lays the foundation for comprehensive comparative genomic studies, including ortholog clustering, accessory genome analysis, structural variation analysis, and gene family expansion and contraction analyses, which will further elucidate the biological novelty, genome evolution, and adaptive mechanisms of different *A. candida* isolates. Additionally, these genomic insights will aid in devising sustainable management strategies to control this pathogen, both in India and globally.

## Data Availability

Data supporting the results of this study are available within the paper. The reagents, plant and microbe materials, and datasets used, generated, and analyzed during this work are available from the corresponding author upon request. The genome sequencing data mentioned in this study has been deposited in the NCBI database under accession number JBBMRL000000000. The Sanger based ITS sequence has been submitted under the NCBI accession PX472090.
