# Supplementary material for: Comprehensive whole genome hybrid assembly unravels genes and secretome of Albugo candida (Indian isolate), causing white rust disease in Brassica juncea
Source: Front Fungal Biol. 2026 Sep 4;7:1756853. doi: 10.3389/ffunb.2026.1756853 (PMC13585763; doi:10.3389/ffunb.2026.1756853)
Supplement: Supplementary file 1 [file Table1.docx]

Supplementary Material

# 1. Supplementary Figures and Tables

## 1.1. Supplementary Figures

**Supplementary Figure 1.** Image showing high–molecular-weight DNA isolated from the white rust (*Albugo candida*) pathogen through the modified CTAB protocol.

## 1.2. Supplementary Tables

**Supplementary Table 1.** Final Reads Selection for hybrid assembly.

| Dataset | Nanopore | Illumina |
| --- | --- | --- |
| Number of sequences | 971643 | 24197250 |
| Sum length | 2423060575 | 3779840898 |
| Minimum length | 826 | 19 |
| Average length | 2493.8 | 156.2 |
| Maximum length | 65583 | 159 |
| Median length | 1842 | 159 |
| N50 | 2937 | 159 |
| Q20(%) | 56.31 | 97.9 |
| Q30(%) | 16.62 | 93.82 |

**Supplementary Table 2.** Statistics of the assembly.

| Dataset | Canu Contigs | Primary Contigs | Alternative Contigs |
| --- | --- | --- | --- |
| Number of sequences | 1205 | 547 | 661 |
| Sum length | 45108710 | 37709860 | 7398850 |
| Minimum length | 1975 | 1975 | 2226 |
| Average length | 37434.6 | 68939.4 | 11193.4 |
| Maximum length | 1031740 | 1031740 | 238463 |
| Median length | 10482 | 23068 | 8506 |
| N50 | 144810 | 182749 | 12141 |

**Supplementary Table 3.** Scaffold statistics of the assembly.

| Dataset | Primary Contigs | Longstitch scaffold | Samba Gapfilling |
| --- | --- | --- | --- |
| Number of sequences | 547 | 415 | 415 |
| Sum length | 37709860 | 37389818 | 37365289 |
| Minimum length | 1975 | 1975 | 1975 |
| Average length | 68939.4 | 90095.9 | 90036.8 |
| Maximum length | 1031740 | 1031740 | 1031740 |
| Median length | 23068 | 18654 | 18654 |
| N50 | 182749 | 306219 | 304373 |
| Ambiguous bases | 0 | 28429 | 3900 |

**Supplementary Table 4.** Racon polishing summary.

| Dataset | racon1 | racon2 | racon3 | racon4 | racon5 |
| --- | --- | --- | --- | --- | --- |
| Number of sequences | 1076 | 1076 | 1076 | 1076 | 1076 |
| Sum length | 44507268 | 44248175 | 43984467 | 43742215 | 43517709 |
| Minimum length | 1646 | 1562 | 1228 | 1161 | 1161 |
| Average length | 41363.6 | 41122.8 | 40877.8 | 40652.6 | 40444 |
| Maximum length | 1032195 | 1032050 | 1030972 | 1030219 | 1029923 |
| Median length | 9614 | 9344 | 9071.5 | 8771 | 8626 |
| Sum gap | 982 | 400 | 400 | 400 | 353 |
| N50 | 253835 | 253257 | 255686 | 255581 | 255938 |

**Supplementary Table 5.** Polca polishing summary.

| Dataset | polca1 | polca2 | polca3 | polca4 | Primary Scaffold |
| --- | --- | --- | --- | --- | --- |
| Number of sequences | 1076 | 1076 | 1076 | 1076 | 415 |
| Sum length | 43521965 | 43521504 | 43521334 | 43521330 | 36886129 |
| Minimum length | 1161 | 1161 | 1161 | 1161 | 1161 |
| Average length | 40447.9 | 40447.5 | 40447.3 | 40447.3 | 88882.2 |
| Maximum length | 1030034 | 1030060 | 1030060 | 1030060 | 1030060 |
| Median length | 8625 | 8624.5 | 8624 | 8624 | 16952.0 |
| Sum gap | 353 | 353 | 353 | 353 | 353 |
| N50 | 255985 | 255990 | 255990 | 255990 | 301912 |
| Substitution Errors | 9008 | 1294 | 582 | 3 | NA |
| Insertion/Deletion Errors | 20477 | 2698 | 1462 | 67 | NA |
| Assembly size | 43517709 | 43521965 | 43521504 | 43521334 | NA |
| Consensus quality | 99.9322 | 99.9908 | 99.9953 | 99.9998 | NA |
| Consensus QV | 31.69 | 40.38 | 43.28 | 57.94 | NA |

**Supplementary Table 6.** Contigs showing no significant similarity to previous reported *A. candida* assemblies.

| S.No. | Contigs | Length |
| --- | --- | --- |
| 1 | contig_4 | 2621 |
| 2 | contig_11 | 2243 |
| 3 | contig_17 | 3529 |
| 4 | contig_22 | 623 |
| 5 | contig_24 | 2723 |
| 6 | contig_25 | 2463 |
| 7 | contig_27 | 2303 |
| 8 | contig_28 | 5245 |
| 9 | contig_29 | 2375 |
| 10 | contig_30 | 2257 |
| 11 | contig_31 | 2994 |
| 12 | contig_32 | 6523 |
| 13 | contig_33 | 4055 |
| 14 | contig_36 | 2266 |
| 15 | contig_37 | 4027 |
| 16 | contig_39 | 7570 |
| 17 | contig_40 | 736 |
| 18 | contig_41 | 3532 |
| 19 | contig_43 | 2897 |
| 20 | contig_47 | 1076 |
| 21 | contig_48 | 841 |
| 22 | contig_49 | 1295 |
| 23 | contig_50 | 2955 |
| 24 | contig_53 | 4494 |
| 25 | contig_55 | 6235 |
| 26 | contig_56 | 2566 |
| 27 | contig_57 | 2449 |
| 28 | contig_58 | 2807 |
| 29 | contig_59 | 2222 |
| 30 | contig_62 | 3304 |
| 31 | contig_74 | 2615 |
| 32 | contig_76 | 3614 |
| 33 | contig_80 | 2385 |
| 34 | contig_83 | 1813 |
| 35 | contig_84 | 1769 |
| 36 | contig_96 | 14427 |
| 37 | contig_99 | 2409 |
| 38 | contig_103 | 10475 |
| 39 | contig_106 | 4497 |
| 40 | contig_108 | 2753 |
| 41 | contig_109 | 2448 |
| 42 | contig_110 | 2407 |
| 43 | contig_117 | 9548 |
| 44 | contig_120 | 542 |
| 45 | contig_121 | 6603 |
| 46 | contig_122 | 1646 |
| 47 | contig_128 | 2261 |
| 48 | contig_129 | 2021 |
| 49 | contig_131 | 2693 |
| 50 | contig_135 | 3624 |
| 51 | contig_137 | 10221 |
| 52 | contig_138 | 3668 |
| 53 | contig_139 | 2240 |
| 54 | contig_146 | 2788 |
| 55 | contig_148 | 2354 |
| 56 | contig_149 | 4376 |
| 57 | contig_150 | 3905 |
| 58 | contig_155 | 2932 |
| 59 | contig_160 | 12017 |
| 60 | contig_163 | 12864 |
| 61 | contig_167 | 2831 |
| 62 | contig_174 | 2358 |

**Supplementary Table 7.** List of primers used for effector validation through qPCR in current study.

| **Seq_id** | **Forword Primer** | **Reverse Primer** |
| --- | --- | --- |
| Ac14696_CRN | GAAGAAATCGGGAAGGAGATGG | GCGTCGTCAGAACTGAGAAA |
| Ac8937_CRN | CCGATCGAAGATACGGCATTTA | AGCCATAGCAGGAAGTTTGG |
| Ac14690_CRN | GTCTCGGACAAAGCTCCATAC | CAGTTGGAACACTTCGCAATTT |
| Ac8954_CRN | GTTTGGGAGACGATTCCTACAT | GACCGATACGCATGTCCATAA |
| Ac7837_CRN | GGAGCTGATCTCTGCGTTAAT | TCGCCTCCCAAACACAAA |
| Ac3931_CRN | GGTTGAGTGAAGGAGAGACAAC | GACTCGGAGGGCATCATTTATT |
| Ac282_ELI | CGAGGATGAGAAGAAGCTCAAA | CTCCAACACGGTAGCATTCA |
| Ac5764_ELI | CGAGGATGAGAAGAAGCTCAAA | CTCCAACACGGTAGCATTCA |
| Ac9701_ELI | GAAAGCATTAGAGCACAGATTCAC | CATACCCGCCGATTCCATATT |
| Ac5718_ELI | GTGAATGCAACATCCCTACAAC | ATCTCCGAGTGCTTTGGATAC |
| Ac6828_ELI | ATCAAACCAAGGGCCGATAG | CGCATTATCCAAGTTGCTTGAG |
| Ac14595_ELI | ATGCGTGCGATATTCCTCAA | GAGAAGATGTCGTAGCCTGATTT |
| Ac6926_CBEL | GTTGTGATGTATGTGCCTCAAC | CTCCTACTTTCCACACCTTCTC |
| Ac11884_CBEL | CTCGTCCAATTTCCTCTCAATCT | GAGGTCATCTCCAGTGAAATCC |
| Ac3297_CBEL | CGCTGTCAATCGACGGAATA | CTGGCAGTACGATTCGGATAAG |
| Ac9163_RxL | GGTGGCTCTACTACTGGAAATC | CCATCACTACCTCCAGAATCATC |
| Ac10281_RxL | GTCCAGCGTCCATGGATAAA | GACATGTAGACGATCAGAACGAG |
| Ac13911_RxL | AGCAAATTGTCCGCCTATGA | CTGGAAGAATGCGCCAAATAAG |
| Ac10898_RxL | CTCCATTCCCTTCCTCCATTTC | TGAACGCCAAGTTGACAGTAT |
| Ac11641_RxL | CTGGACTTATCTGGCTTCTAGTG | CGCAAGGAACTATCAGGGTATC |
| Ac9688_RxL | GGAGTAGCGTTGGATTGAATTG | GTAGACTGAGACCGCAGTAATC |
| Ac9891_RxL | TCTTCCAATCAGTTCCGACTTT | CTTCTCCGTTATATCGCATAGCA |
| Ac1212_RxL | CTCACTCGTGGAAGCGATATG | GGTGCCTGTGATCAGCTTTA |
| Ac5570_RxL | CTCAATGCTGACATTCCACAATC | GCCAATGTCTGATCCGTTACA |
| Ac12847_RxL | TGGAGAAGGTGAGGTTTGATG | CGAGGATGGTCGATATAGGTTTAC |
| Ac13293_RxL | ACAGAATTATCCGCCAGAAGG | GTAGTACGAGCAACTTGGAGAC |
| Ac4413_RxL | GGCGTATGGAAGAAGGAAT | CCGGAGCAAGCATCATCAA |
| Ac9130_RxL | GATTGGTATTTGCCGCATCCG | GCAAACTCCAATTCAGATTGG |
| Ac8380_CCG | CCACCTCCACCTGAATGTAAT | CGAACTTTGCGCGTCTTT |
| Ac1703_CCG | ACGCTACGGGAAGGAAATG | TGGCAGATCGAAGCAAGAG |
| Ac13392_CCG | GGGCGTGATTTACGAAACTG | GAGGGACTTCGTCGGTTAAT |
| Ac6650_CCG | CCGATGACAATGAGCCTGTATG | TGGATGGATCGACGGAGTATAG |
| Ac10288_CCG | AGGTAGAGGTCGCTCAGATT | CCCAGTACAAGGTTAGCTCTTT |
| Ac9641_CCG | GGGTGCATCACACTGGATAA | CACTATACGACTTGCGACTCTAC |
| Ac11046_CCG | CAGAAAGGCAGAGACCTCATC | GCAGCCAAGCATCCATATTTC |
| Ac12539_CCG | TGCGGATGTCGAGATTATGC | TAGATTGAGAAGGCAACAAGGG |
| Ac1330_CCG | CGTCTGCCACAGCAATACT | GGACTTACCCTGACACTCAATC |
| Ac1549_CCG | CATCGTGTCATCGGTGTCTT | TGCCGTCCATTGAGAAAGTAG |
| Ac2276_CCG | GCCTCTGATAGACGGACTATTG | CACCTATACAGCGTGGAGTTT |
| Ac_10587_CCG | TATGTACTGCACGGCGAATG | CCACCTTCCGGAATGAGAATAC |
| Ac9267_CCG | GAGTCCGAAGAAGCGGATTT | ACACCTTCTTGTGGTGGTATG |
| Ac10212_CCG | GGCACAGAGAAAGAAGGAGAAG | GAACTCCGAACGATGAGCTATG |
| Ac10267_CCG | AGACGTCATGAGCTGTCATTC | GTGTCGTGATGGGACCTTTAATA |
